# Positively selected genes in the hoary bat (*Lasiurus cinereus*) lineage: prominence of thymus expression, immune and metabolic function, and regions of ancient synteny

**DOI:** 10.7717/peerj.13130

**Published:** 2022-03-17

**Authors:** Robert S. Cornman, Paul M. Cryan

**Affiliations:** Fort Collins Science Center, U.S. Geological Survey, Fort Collins, CO, United States of America

**Keywords:** Hoary bat, Adaptation, Positive selection, Immunogenetics, Thymus, Conserved synteny, Cat-eye critical region, Tbx gene family, Chrna9

## Abstract

**Background:**

Bats of the genus *Lasiurus* occur throughout the Americas and have diversified into at least 20 species among three subgenera. The hoary bat (*Lasiurus cinereus*) is highly migratory and ranges farther across North America than any other wild mammal. Despite the ecological importance of this species as a major insect predator, and the particular susceptibility of lasiurine bats to wind turbine strikes, our understanding of hoary bat ecology, physiology, and behavior remains poor.

**Methods:**

To better understand adaptive evolution in this lineage, we used whole-genome sequencing to identify protein-coding sequence and explore signatures of positive selection. Gene models were predicted with Maker and compared to seven well-annotated and phylogenetically representative species. Evolutionary rate analysis was performed with PAML.

**Results:**

Of 9,447 single-copy orthologous groups that met evaluation criteria, 150 genes had a significant excess of nonsynonymous substitutions along the *L. cinereus* branch (*P* < 0.001 after manual review of alignments). Selected genes as a group had biased expression, most strongly in thymus tissue. We identified 23 selected genes with reported immune functions as well as a divergent paralog of *Steep1* within suborder Yangochiroptera. Seventeen genes had roles in lipid and glucose metabolic pathways, partially overlapping with 15 mitochondrion-associated genes; these adaptations may reflect the metabolic challenges of hibernation, long-distance migration, and seasonal variation in prey abundance. The genomic distribution of positively selected genes differed significantly from background expectation by discrete Kolmogorov–Smirnov test (*P* < 0.001). Remarkably, the top three physical clusters all coincided with islands of conserved synteny predating Mammalia, the largest of which shares synteny with the human cat-eye critical region (CECR) on 22q11. This observation coupled with the expansion of a novel *Tbx1*-like gene family may indicate evolutionary innovation during pharyngeal arch development: both the CECR and *Tbx1* cause dosage-dependent congenital abnormalities in thymus, heart, and head, and craniodysmorphy is associated with human orthologs of other positively selected genes as well.

## Introduction

Bats are a highly diverse clade of mammals exhibiting well-known innovations such as flight, echolocation, and extremes of metabolism and longevity. They have important ecological roles including pollination, seed dispersal, and insect predation, and as a group are understudied and threatened (Adams & Pedersen, 2013). Recent intensification of stresses in North America related to white-nose syndrome (Hoyt, Kilpatrick & Langwig, 2021) and wind-turbine fatalities (Frick et al., 2017) have heightened the need for research to support bat management, yet many species are challenging to census and study.

A species of growing concern for U.S. management agencies (Hein, Hale & Straw, 2021) is the hoary bat (*Lasiurus cinereus*), a tree bat comprised of two disjunct populations in North America and South America. Individuals are highly migratory, traveling thousands of kilometers per year in search of prey or mates (Cryan, Stricker & Wunder, 2014). Little is known about their hibernacula or the physiological character of hibernation in the species, and while the effective population size has historically been large (Korstian, Hale & Williams, 2015; Sovic, Carstens & Gibbs, 2016; Pylant et al., 2016; Cornman et al., 2021), evidence of range contraction (Rodhouse et al., 2019) and high turbine-associated mortality (Frick et al., 2017) suggest population pressures are currently high. Importantly, wind-turbine mortality has a strong behavioral component as individuals actively approach the moving blades (Cryan et al., 2014) for reasons that remain unclear. It has been hypothesized that the turbines mimic a natural stimulus (*e.g*., Corcoran & Weller, 2018) and technologies to counter this attraction are being explored (*e.g*., Weaver et al., 2020). Interestingly, a novel microcall has recently been identified from acoustic analysis of *L. cinereus* echolocation (Corcoran & Weller, 2018), although the phylogenetic distribution and consistency in form of this behavior remain to be fully determined. Elucidating the evolutionary timing and developmental or physiological bases of these and other distinctive traits can help researchers better understand how ecological niches evolve (*e.g*., Oliver et al., 2010; Zhang et al., 2019b; Davies et al., 2020) and potentially their sensitivity to perturbation. Genes underlying recent physiological adaptations could be particularly relevant as biomarkers of organism condition, and standing variation at such loci may influence future adaptation to anthropogenic pressures (Bitter et al., 2019).

Whereas genomic resources have begun to appear for the hoary bat in support of population analyses (Pinzari et al., 2020; Cornman et al., 2021), evolutionary comparisons with related bat genomes have not yet been explored. While we do not propose specific hypotheses to be addressed, descriptive assessments of protein-evolutionary rates provide the groundwork that could help define future hypotheses on the molecular basis of adaptation in the hoary bat lineage. Indeed, comparative genomics has revealed molecular mechanisms of adaptation, novelty, and constraint across diverse evolutionary clades (Kosiol et al., 2008; Corradi et al., 2010; Manceau et al., 2010; Lindblad-Toh et al., 2011; Roux et al., 2014; Neafsey et al., 2015; Hughes et al., 2018; Beichman et al., 2019). The Bat1K project (Teeling et al., 2018), for example, has begun to produce insights into bat evolutionary genomics (Jebb et al., 2020).

In this study, we annotated a preliminary gene set based on protein homology and performed branch-specific tests of evolutionary rate of coding sequences (the estimated ratio of nonsynonymous substitutions to synonymous substitutions). We found a large number of significant tests after manual review of initially significant alignments. Searches of gene annotation databases and the literature for human and mouse orthologs identified several major themes including immune function, metabolic homeostasis, and mitochondrial localization. Available gene expression data for mouse orthologs indicated biased expression of positively selected genes in the thymus, a key site of adaptive immunity (Thapa & Farber, 2019). Three physical clusters of positively selected genes were noted, all of which coincide with genomic islands of conserved gene content and order (ancient synteny) predating Mammalia; two of these date to the origins of Tetrapoda. The largest cluster of positively selected genes was syntenic with the human cat-eye critical region (CECR) on 22q11, which is associated with a spectrum of congenital developmental disorders (Footz et al., 2001). The genes we identified are candidates for further vetting by genome sequencing of congeners and other close relatives, comparative transcriptomics, and intraspecific polymorphism scans.

## Materials and Methods

### DNA extraction and sequencing

Genome sequencing and assembly are detailed in Cornman et al. (2021). Briefly, blood was drawn from an adult female specimen within 12 h *post mortem* and shipped cold to MedGenome Inc. for DNA extraction and 10X linked-read library preparation. Sequencing was performed on the Illumina NovaSeq platform in a paired 150-bp layout. A total of 594,097,777 read pairs were generated together with the associated linking index reads (available from the National Center for Biotechnology Information (NCBI) under BioProject accession PRJNA601154). Two iterations of linked-read assembly were performed with the supernova assembly program on the Yeti supercomputing resource of the U.S. Geological Survey (https://www.usgs.gov/core-science-systems/sas/arc/usgs-yeti-supercomputer). The final diploid assembly had coverage metrics in line with manufacturer recommendations. Two ‘pseudohaploid’ sequences, between which unphased haplotype blocks are arbitrarily split (see Weisenfeld et al., 2017 for details), were deposited in NCBI’s Genome database (accessions GCA_011751065.1 and GCA_011751095.1). Of the two reference pseudohaplotypes, we arbitrarily chose to annotate the pseudohaplotype associated with accession GCA_011751065.1. However, our analysis was performed on the original scaffolds output by the supernova assembly program (available from Cornman et al., 2020), not the slightly more fragmented assembly generated during submission to NCBI. Fragmentation occurs because NCBI policy requires scaffolds be split on strings of 10 or more ambiguous bases when the gap size is not explicitly estimated, but this threshold may not be a strong indication of assembly ambiguity in linked-read data.

### Gene annotation

Annotation was performed with Maker v. 2.31.10 (Cantarel et al., 2008). Repeat masking was performed within Maker using a licensed RepBase database (v. 24.12; Jurka et al., 2005) and RepeatMasker (Tarailo-Graovac & Chen, 2009) database Dfam3.1, in addition to *de novo* repeat discovery. As no transcriptomic data were available for *L. cinereus*, predictions were based on protein homology only, using all Chiroptera identical protein groups (downloaded from NCBI on January 29, 2020) as the reference database. Diamond (Buchfink, Xie & Huson, 2015) was selected as the alignment package and exonerate v. 2.2.0 (Slater & Birney, 2005) was selected to create homology-based gene models. A total of 20,663 protein models were generated (Cornman & Cryan, 2022), which is comparable to the 19,728 coding models and 1,713 pseudogenes listed in the Ensembl genome database (Yates et al., 2020) for *M. lucifugus*. The predicted proteins included 84.9% of ‘universal’ mammalian orthogroups, as assessed with Busco 5.1.3 (Seppey, Manni & Zdobnov, 2019) using OrthoDB version 10 (Kriventseva et al., 2019), of which 98.7% were complete and single copy. In comparison, 96% of the same benchmark set were detected for the related vespertilionid bat *Eptesicus fuscus* (see below), of which 99.6% were complete and single-copy. We did not annotate non-coding features such as non-coding RNAs, transfer RNAs, or ribosomal RNAs. Gene predictions in GFF format along with the corresponding coding sequences are available from Cornman & Cryan (2022).

### Evolutionary rate analysis

RefSeq mRNA models and their protein translations were downloaded from NCBI for *M. lucifugus* (assembly accession GCA_000147115.1; Suborder Yangochiroptera, Family Vespertilionidae) (Lindblad-Toh et al., 2011) and six other bat species (Jebb et al., 2020): *E. fuscus* (assembly accession GCA_000308155.1, EptFus1.0; Yangochiroptera, Vespertilionidae), *Hipposideros armiger* (assembly accession GCA_001890085.1, ASM189008v1; Dong et al., 2016; Yinpterochiroptera, Hipposideridae), *Phyllostomus discolor* (assembly accession GCA_004126475.3 mPhyDis1.pri.v3; Yangochiroptera, Phyllostomidae), *Pteropus vampyrus* (assembly accession GCA_000151845.2 Pvam_2.0; Yinpterochiroptera, Pteropodidae), *Rhinolophus ferrumequinum* (assembly accession GCA_004115265.2, mRhiFer1_v1.p; Yinpterochiroptera, Rhinolophidae), and *Rousettus aegyptiacus* (assembly accession GCA_014176215.1 mRouAeg1.p; Yinpterochiroptera, Pteropodidae). These accessions represent most full-genome derived proteomes available for bats at the time of analysis, after excluding closely related species such as *Myotis* congeners. Sequences were filtered on locus name to include only the first listed isoform when multiple isoforms of a locus were present.

To identify orthologous groups of sequences and generate nucleotide alignments for analysis of substitution rates, we adapted the workflow of Santagata (2021). Orthologous groups were first identified using Orthofinder (Emms & Kelly, 2019) and filtered to include only orthogroups with a single representative from every species and at most a single paralog in one species other than *L. cinereus*. The number of “perfect” single-copy, complete orthogroups identified was 9,064, with an additional 466 “near-perfect” orthogroups with a single paralog in a species other than *L. cinereus*. Only perfect or near-perfect orthogroups were used because closely related paralogs can evolve non-independently by gene conversion, neofunctionalization, or subfunctionalization (Casola, Ganote & Hahn, 2010; Sandve, Rohlfs & Hvidsten, 2018), distorting the estimate of background evolutionary rate. Orthogroups lacking orthologs in any species were also excluded due to the reduced power to estimate evolutionary rate.

Near-perfect orthologous groups were trimmed of the inferred paralog using phylopypruner (Thalén, 2018). Orthologous protein sequences were aligned with mafft (Katoh et al., 2002). As protein-coding models occasionally contain stop codons (*e.g*., due to annotation error or evidence of stop codon read-through), we converted in-frame stop codons to ambiguity characters. This conversion was performed because mafft treats stop characters in protein coding sequence as gaps, which can result in a loss of correspondence between protein and nucleotide sequences. Nucleotide alignments were reconstituted from protein alignments and formatted for PAML using pal2nal (Suyama, Torrents & Bork, 2006). Orthogroups with sequences less than 100 amino acids in length were discarded as too short for analysis.

Evolutionary rate analysis assumes that the gene tree has the same topology as the species tree and paralogs and orthologs are not conflated. It further assumes orthology of each aligned codon, which may be uncertain for some parts of an alignment. Error can arise from poorly conserved or low-complexity regions, incorrect 5′ or 3′ gene termini, missed exons, and frameshift errors in the reference genome. Gross errors are particularly likely at alignment edges because the corresponding exons may have low coding content and thus more likely to be missed by protein-alignment methods. We therefore trimmed alignments from both the 5′ and 3′ ends until the first completely conserved amino-acid triplet was observed in each direction. This approach is likely conservative, entailing a tradeoff between alignments that are shorter and enriched in relatively well-conserved protein regions versus alignments containing more errors and requiring greater manual evaluation. While there is a clear need to mask ambiguous sequence alignments that bias evolutionary rate estimation, methods that balance this need against the loss of informative sites remain an area of active investigation (Di Franco et al., 2019). For example, Beichman et al. (2019) manually reviewed over 600 candidates for positive selection after applying four such methods, resulting in only 18 accepted tests, and found that different masking approaches gave divergent results. We therefore followed Jebb et al. (2020) in using the T-Coffee alignment consistency score as a guideline for validating all manually revised alignments as well as all alignments with a significant PAML result (see below), requiring a minimum score of 950 (the maximum possible score is 1,000) (Notredame, Higgins & Heringa, 2000; Chang, Di Tommaso & Notredame, 2014). Similar considerations led us to filter orthogroup alignments based on the tree length (substitutions per codon) calculated by PAML: we excluded 134 orthogroups with tree lengths greater than 10, as by inspection these outliers were highly enriched in alignment error (Supplemental File S1).

We used the codeml program of PAML 4.9i (Yang, 2007) to test for positive selection acting on the *L. cinereus* branch of the multilocus species tree estimated by Orthofinder, after unrooting the Orthofinder-inferred tree with the R package *ape* (Paradis, Claude & Strimmer, 2004). PAML models 1 and 2 were first fit to the tree topology assuming all rate classes applied to all branches. Model 1 assumes a fraction of sites p_0_ have evolutionary rate ω_0_ < 1 (purifying selection), with the remaining sites evolving neutrally (ω_1_ = 1). Model 2 estimates two evolutionary rates and two proportions, dividing sites into those evolving under purifying selection (ω_0_ < 1) or positive selection (ω_2_ > 1), with the balance evolving under neutrality (ω_1_ = 1). Model 2 was then re-fit after designating the *L. cinereus* branch as the foreground branch with a potentially different value of ω_2_ than the background branches (for which ω_2_ is fixed at 1). For each pair of models fit to a given alignment, twice the difference in the natural log of the reported likelihoods constitutes a test metric that can be compared to the χ^2^ probability distribution (Yang, 2007) with one degree of freedom. Although Model 1 is not the recommended null model for detecting branch-specific selection, we estimated its likelihood to confirm it was not appreciably greater than Model 2 estimates, which might suggest poor estimate convergence.

Initially significant alignments were manually checked for misaligned or low-complexity sequence. Gapped low-complexity regions were deleted between the nearest 5′ and 3′ conserved codons. Runs of amino-acid substitutions unique to a particular branch were considered errors in the predicted primary sequence and were also deleted between the nearest 5′ and 3′ conserved codons. Gross misalignments were not reanalyzed if little nonsynonymous variation was evident on the *L. cinereus* branch and were discarded as false positives. Because of the need for manual masking of poorly aligned regions, resulting in many initial test metrics being judged invalid on inspection or requiring a revised test (see “Results”), the standard approach to false discovery correction is unsatisfactory. This is because the orthogroups with highest test statistics are enriched in alignment error: ranking test results by test statistic and revising the significance threshold based on test number would therefore have resulted in their preferential acceptance at the expense of orthogroups with more biologically plausible test statistics. We addressed the lack of a straightforward correction procedure by setting a relatively strict α = 0.001 for the likelihood ratio test. Summary metrics and test results for the 9,447 groups of orthologous genes (“orthogroups”) identified by the workflow are provided in Supplemental File S1. Gene annotations, initial orthogroup alignments, and manually revised orthogroup alignments are provided in Supplemental Files S2–S4.

We compared PAML results with those of the ‘adaptive’ branch-specific model implemented by the program aBSREL of the HyPhy package (Smith et al., 2015). This program tests whether a foreground branch is evolving under positive diversifying selection while allowing separate background rates to potentially be calculated for other branches in the tree. This is potentially a more parameter-rich model depending on the outcome of the adaptive algorithm, with each additional parameter necessarily estimated from fewer data. The aBSREL method was developed for larger alignments such as viral data sets (Smith et al., 2015) and is only used here to check for divergent behavior of the two methods. For our objective of identifying loci positively selected in the *L. cinereus* lineage and given the relatively small number of sequences available per locus, we believe estimating a single rate of purifying selection from all background branches with PAML is the preferred abstraction because more data are used to estimate that parameter and contrast it with the *L. cinereus* rate.

*P*-values estimated by PAML were generally lower (more significant) than those calculated by aBSREL (Supplemental File S5). The two methods were often concordant but exhibited a qualitative pattern in that aBSREL *P*-values were either low and log-linear with PAML *P*-values or much higher and unrelated to the PAML *P*-values. This duality was most striking for longer alignments (Supplemental File S5), whereas there was little concordance between *P*-values estimated by the two methods for the shortest alignments. The dual distribution presumably reflects the extent to which the adaptive aBSREL algorithm attributes evolutionary change to background rather than foreground branches of the phylogeny (a single background rate is assumed by PAML). If some background branches are estimated by aBSREL to have relatively high evolutionary rates, the hypothesis of selection on the foreground branch (*L. cinereus*) should be correspondingly less supported, particularly as alignment lengths decrease.

For each tested orthogroup, the *M. lucifugus* RefSeq protein ID was used to extract the corresponding NCBI Gene database record (Brown et al., 2015), which was parsed to obtain the mouse and human ortholog identifiers, when available. We used the DAVID functional annotation tool (Huang et al., 2007), accessed 12/21/2020, to test for significant enrichment, using the default categories implemented for mouse. From the XML-formatted entries of the NCBI Gene database, we also extracted gene names, gene symbols, summary comments, and expression data for 30 mouse tissues from the MODENCODE project (Yue et al., 2014). To avoid repeated tests of tissues with highly similar expression profiles, we calculated all pairwise Pearson correlations between tissues and randomly removed one tissue of each pair having a correlation coefficient above 0.90, resulting in the exclusion of five tissues. Including highly correlated tissues would have falsely inflated the apparent number of independent tests, thereby making false discovery corrections too severe.

The cumulative distribution function of genes in consecutive windows was computed with the ecdf function in R (R Core Team, 2019) and the observed distribution of significant genes compared to this background expectation using the discrete Kolmogorov–Smirnov test of Dimitrova, Kaishev & Tan (2020). *L. cinereus* genes were assigned genomic coordinates based on the midpoint of the gene model, and tabulated in consecutive windows of 1 MB, with the final window on each scaffold truncated in length based on the residual sequence (genes annotated on short scaffolds less than 1 MB in length were excluded from this analysis). Scaffolds were ordered arbitrarily by name as this has no bearing on the test outcome. Rejection of the null hypothesis indicates that the observed distribution of significant genes differs from expected, but does not specify which genes contribute to the divergence.

Phylogenetic analyses were performed with MegaX (Kumar et al., 2018). Synteny plots were based on protein similarity and computed with Mummer v.4.0.0 (Marçais et al., 2018), with a seed length of 35 and nonunique matches filtered. Protein secondary structure was predicted with TMHMM v. 2.0 (Sonnhammer, von Heijne & Krogh, 1998).

## Results and discussion

### Genes under positive selection

Evolutionary rates of predicted *L. cinereus* coding sequences and their inferred orthologs in seven other bat species (orthogroups) were estimated with PAML. Of 9,447 orthogroups tested, 487 were initially significant and further reviewed manually, of which 90 were accepted as free of obvious errors affecting the *L. cinereus* branch. An additional 78 alignments were discarded because they exceeded a nonsynonymous tree-length threshold of 10 (see “Materials and Methods”), whereas 105 alignments were discarded on the basis of evident errors, with insufficient nonsynonymous variation in credible regions of the alignment to justify re-analysis. Gross alignment errors typically manifested as a combination of long gaps and runs of divergent residues unique to a single taxon that would be more parsimoniously explained by errors in the primary sequence prediction. In a few instances, the *L. cinereus* gene appeared to be paralogous based on *post hoc* BLASTP searches against all Chiroptera proteins in the *nr* database. Those searches revealed the *L. cinereus* sequence to be more similar to other bat proteins not included in the study than to sequences in the assigned orthogroup cluster. Errors of paralogy presumably arise because the true ortholog was lost or unannotated in *L. cinereus*. The remaining 214 initially significant alignments were manually trimmed and re-analyzed; of these, the *L. cinereus*-specific positive selection model was accepted for 60. One of these 60 significant orthogroup alignments (OG0012876, see Supplemental File S1) had a revised alignment length of 99 codons, below our 100-codon threshold for initial tests, but we chose not to exclude it from the second round of manual testing because the full protein was only slightly longer (102 amino acids) and invariant in the other seven species, whereas the *L. cinereus* sequence had four nonconservative substitutions not present in any other bat BLASTP protein match in *nr*.

In total, 150 tested alignments (1.6%) were considered significant, which is relatively high compared to some studies in other mammals. For example, Beichman et al. (2019) identified 18 positively selected genes in the ancestor of sea otters and Kosiol et al. (2008) identified a total of 144 significant genes across eight different branch-specific tests of mammalian genomes (*i.e*., an average of 18 unique genes per mammalian lineage tested). On the other hand, Shen et al. (2010) identified 100 positively selected genes in the lineage leading to bats with other mammals as the background, and Hawkins et al. (2019) identified 181 positively selected genes from a combined analysis of diverse bat genomic and transcriptomic sequence. Thus, our results further support high rates of adaptive protein evolution in bats relative to other mammalian clades (but see Jebb et al., 2020).

For 25 of the 150 genes identified as under positive selection, the evolutionary rate ω_2_ of positively selected genes was estimated to be one rather than greater than one. The average length of these 25 alignments was substantially shorter than the other 125 (455.6 *vs* 638.5 codons, respectively). While genes with ω_2_ estimates of one may be false positives that are actually evolving neutrally, ω_2_ estimates strongly trade off with the estimated proportion of sites (p_2_) to which the rate applies (Supplemental File S6), suggesting that the joint estimation of these two parameters is challenging. Furthermore, a comparable number of genes (29) had effectively infinite ω_2_ estimates (*i.e*., the programmatic maximum of 999), which seems no more realistic than an ω_2_ estimate of one. We therefore considered only the overall model likelihoods in detecting positive selection and did not further require that ω_2_ estimates exceed some threshold. Of course, we did require that the ω_2_ estimate not be less than one, which is unlikely but formally possible even when the positive selection model is accepted (Yang, 2007).

We found a significant association between single-exon genes and the frequency of positive selection (*P* = 4.28E−5, Fisher’s 2 × 2 exact test with df = 1; Supplemental File S7). A cautious view of this finding is that genes with high evolutionary rates are enriched in weakly constrained retrogenes, which are initially single-exon and may be unexpressed. However, transcript and protein expression data are ultimately required to evaluate gene activity and the evolutionary dynamics of retained retrogenes has been shown to be shaped by diversifying and purifying selection mechanisms (Vinckenbosch, Dupanloup & Kaessmann, 2006; Gayral et al., 2007). Thus, neutral evolution is not necessarily expected for retrogenes, and in any event the statistical test we performed rejects a hypothesis of neutral evolution in favor of adaptive evolution. We also note that the number of single-exon genes in *L. cinereus* may be methodologically inflated, because noncoding exons are not readily predictable based on protein homology alone. Single-exon genes do generally appear to have higher evolutionary rates than multiexon genes regardless of these ascertainment issues (Shabalina et al., 2010), so the statistical association we observed is consistent with multiple causes.

**Figure 1:**
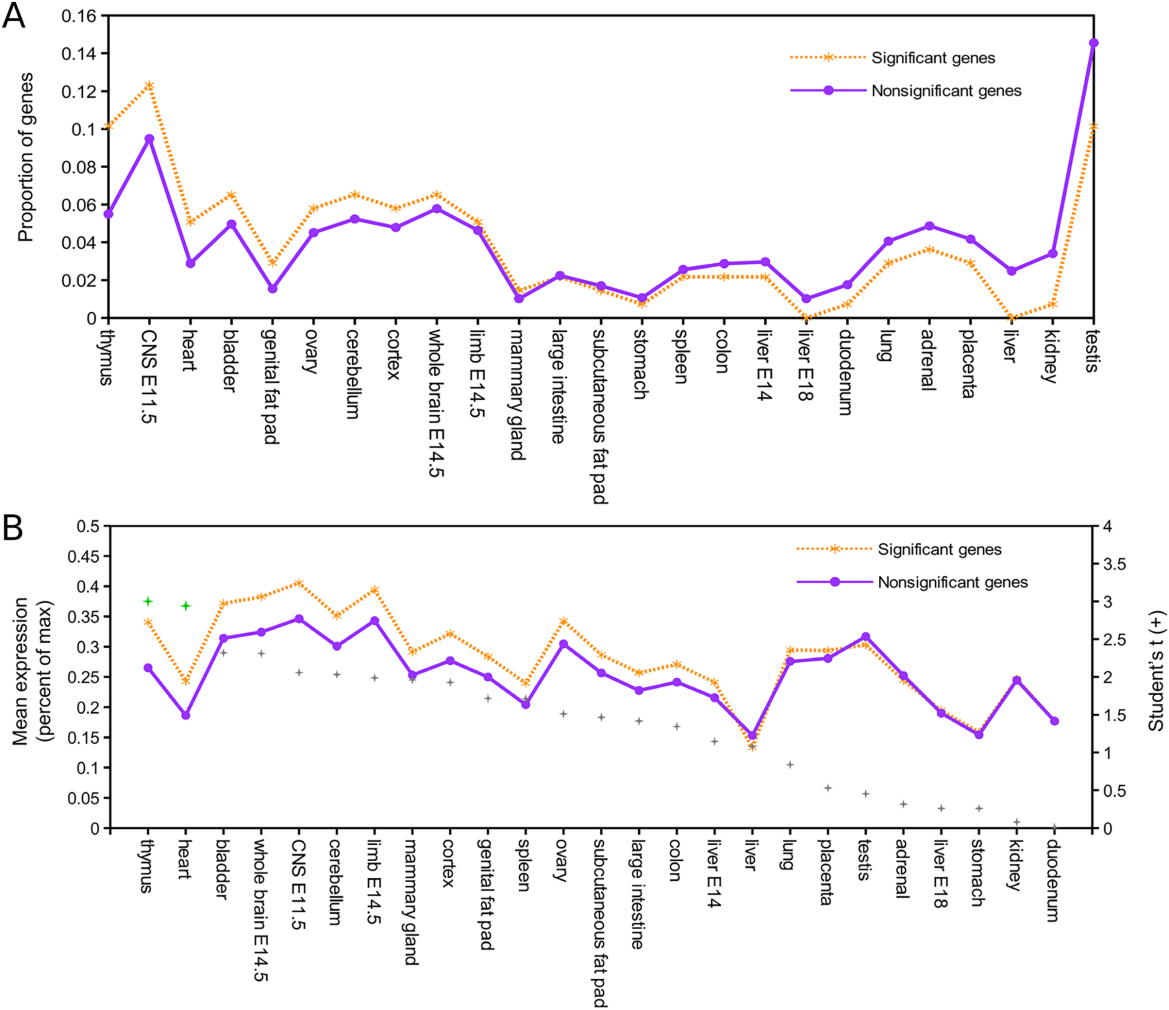
Genes positively selected in the *Lasiurus cinereus* lineage have biased expression in the mouse thymus. (A) Proportion of mouse orthologs of selected genes that have peak relative expression in each tissue. The proportion is significantly higher in thymus for positively selected genes than for other genes when all other tissues are binned together (see text for details). (B) The mean relative expression of positively selected genes is highest in thymus. The difference in means is significant at *P* < 0.05 (Student’s *t*-test) for both thymus and heart after Benjamini–Hochberg correction for multiple tests.

Relative expression data were available for 137 of the 143 mouse orthologs associated with positively selected genes and for 8,410 of the remaining orthogroups (Supplemental File S8). Figure 1A shows the proportion of genes in each of these two groups that have peak expression in the corresponding tissue, sorted by the intergroup difference. Figure 1B shows the average expression of genes in each group (normalized to their maximum) by tissue type, sorted by Student’s t statistic for the corresponding difference of means. By both standards, the largest difference in expression between significant and nonsignificant genes was in thymus tissue. After pooling all other tissues into a single bin for comparison, Fisher’s exact test confirmed a significantly higher incidence of peak expression in thymus for positively selected genes (*P* = 0.023). Mean expression of positively selected genes was significantly higher than nonselected genes in both thymus and heart (two-sided *t*-test with equal variance assumed, *P* < 0.05) after Benjamini–Hochberg correction for multiple tests. Biased expression in thymus of positively selected genes suggests positive selection on immunodevelopment, as the thymus is the site of T cell maturation (reviewed by Thapa & Farber, 2019), defects of thymus development are associated with immune dysfunction (*e.g*., Bockman, 1997), and immunosenescence is associated with regression of the thymus with age (Palmer, 2013; Rezzani et al., 2014).

### Genomic clusters of significant genes

**Figure 2:**
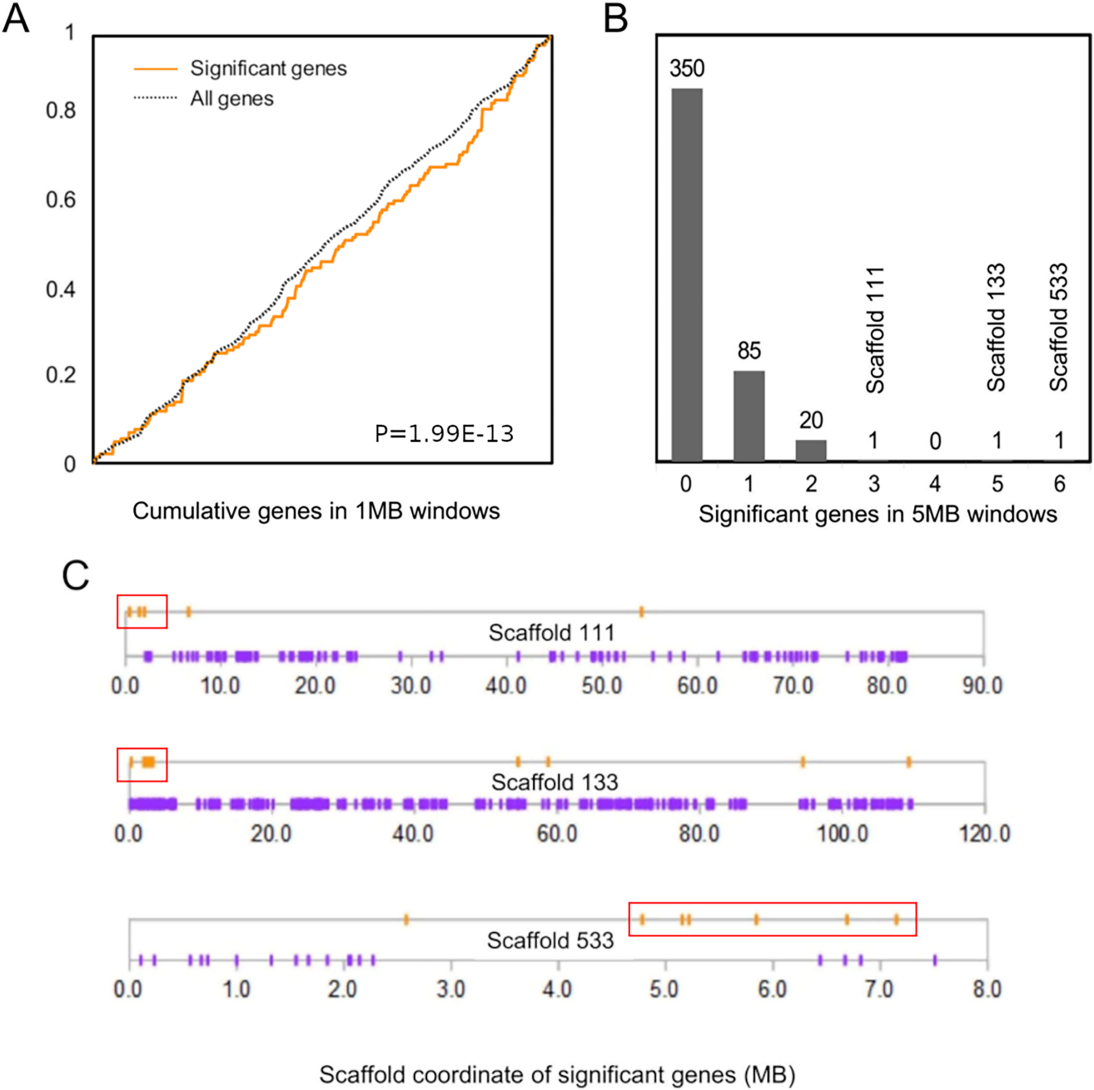
Positively selected genes are clustered in the *Lasiurus cinereus* genome. (A) The cumulative distribution of significant genes along scaffolds differs significantly from that of all tested orthogroups by discrete Kolmogorov–Smirnov test. (B) Distribution of the counts of significant genes in consecutive 5-Mb windows. The scaffold locations are shown for the three windows with three or more significant genes. (C) Schematic of the distribution of significant genes (orange marks) and nonsignificant genes (purple marks) by coordinate position on the three scaffolds marked in (B). Red boxes indicate the selected genes within 5-Mb windows marked in panel (B).

The cumulative distribution of significant genes along scaffolds (Fig. 2A) differed from that expected based on the distribution of all genes (*P* = 1.99E−13 by discrete Kolmogorov–Smirnov test). To identify clusters of significant genes that likely contributed to this result, we tabulated the number of significant genes in 5-Mb windows and identified three windows containing three or more selected genes (Fig. 2B). Interestingly, all three clusters are near the end of their respective genomic scaffolds (Fig. 2C), although it remains to be confirmed whether they are actually subtelomeric in *L. cinereus*. One cluster contains five significant genes with midpoints in a 2-Mb window, situated near the terminus of scaffold 133, which at 109 Mb in length is the longest scaffold of the assembly. A second cluster contains three genes with midpoints within a 2-Mb window near the end of another long scaffold, scaffold 111. A third cluster contains six genes with midpoints within a 3-Mb window on scaffold 533. Two of these three clusters of positively selected genes lie in genomic regions of conserved synteny with other tetrapods, whereas the third is syntenic with other mammals (Supplemental File S9). While regions of conserved synteny are expected due to the variable persistence of ancestral vertebrate linkage groups (Simakov et al., 2020), the overlap between positive selection and conserved synteny in *L. cinereus* is striking and suggests that selection may have targeted co-evolving genes maintained in syntenic blocks. Factors hypothesized to favor the maintenance of synteny include co-regulated expression *via* local chromatin structure or shared promoter elements (Reimegård et al., 2017; Rokas et al., 2018) and epistatic effects of multigene haplotypes on fitness (“co-adapted gene complexes”, *e.g*., Dobzhansky & Pavlovsky, 1958; Michalak, 2008; Hittinger et al., 2010).

The cluster of positively selected genes on scaffold 133 includes orthologs of the mouse genes *Fam173a*, *Lmf1*, *Tsr3*, *Cramp1l*, and *Ndufb10* (Supplemental File S10). Three of the five genes have roles at the nexus of redox homeostasis and metabolism, as *Fam173a* and *Ndufb10* are both mitochondrial membrane proteins that affect rates of mitochondrial respiration and *Lmf1* is generally responsive to redox stress as it relates to membrane-protein folding. Specifically, *Fam173a* encodes a mitochondrial lysine-specific methyltransferase that targets adenine nucleotide translocase (Małecki et al., 2019), and NADH dehydrogenase (ubiquinone) 1 beta subcomplex 10 (*Ndufb10*) is an accessory subunit of respiratory complex I important for complex assembly (Friederich et al., 2017). *Lmf1* (lipase maturation factor 1) is important for regulating plasma lipid levels and responds to redox stress on the endoplasmic reticulum (ER) (Mao et al., 2014). It functions as a molecular chaperone enabling proper folding and activity of ER membrane proteins. The other two genes in the cluster on scaffold 133 do not share an apparent functional association with the previous three genes. *Cramp1l* encodes a chromatin remodeling domain well conserved in vertebrates. *Tsr3* (20S rRNA accumulation factor) contributes to the maturation of 18S rRNA *via* the post-transcriptional modification of a conserved pseudouridine-like nucleotide (Meyer et al., 2016).

**Figure 3:**
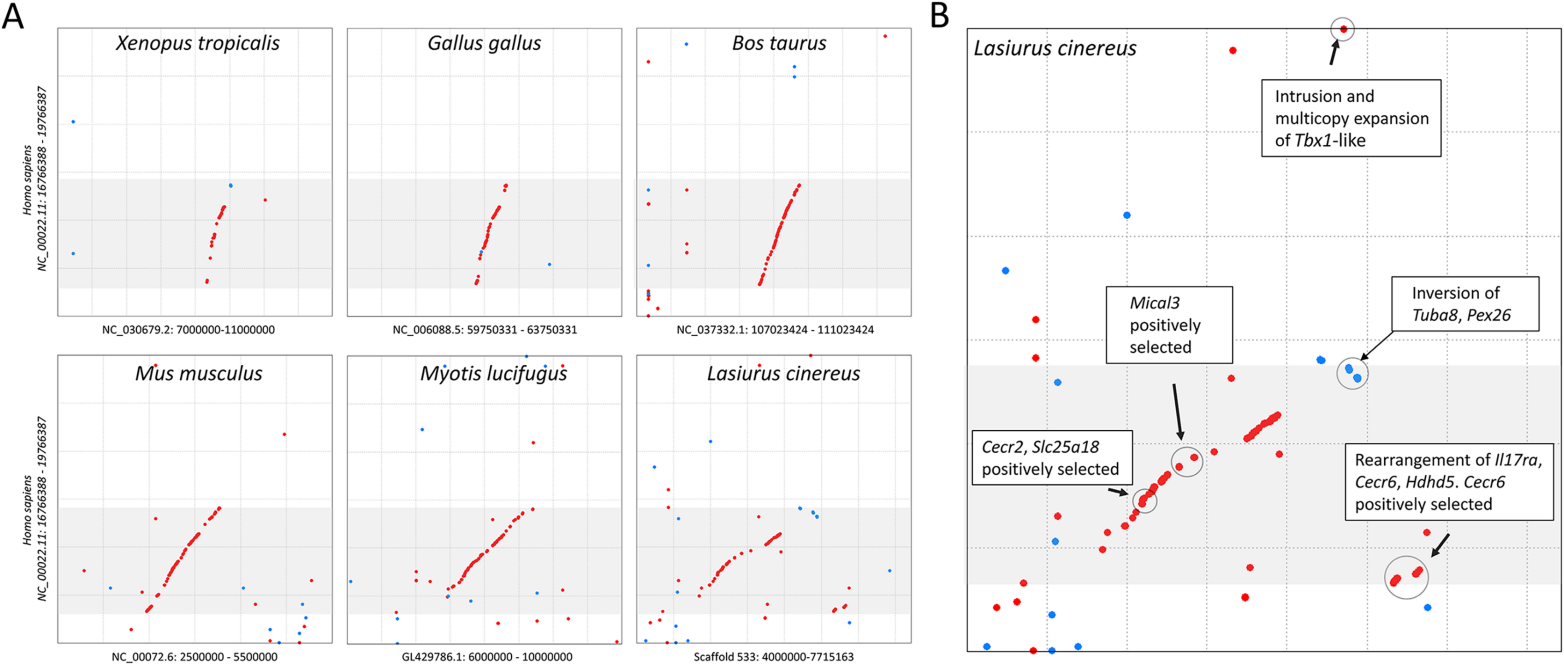
Positively selected genes clustered on scaffold 533 are syntenic with the human cat-eye critical region (CECR). (A) Protein-based alignment between human chromosome 22 in the vicinity of the CECR (gray-shaded region) and six other species. Red and blue dots indicate homology in the same and reverse orientations, respectively. (B) Positively selected genes and structural variation in the syntenic region in *Lasiurus cinereus*.

The cluster of positively selected genes on scaffold 533 includes *Rps13*, *Cecr2*, *Slc25A18*, *Mical3*, and *Cecr6*. All but the first listed of these genes are landmarks of the cat-eye critical region (CECR) on human 22q11 that is syntenic with other mammals (Puech et al., 1997; Footz et al., 2001). Alignments of the human region to scaffold 533 and scaffold GL42786.1 of *M. lucifugus* confirm the region is syntenic with bats as well (Fig. 3). Synteny is also shared with other tetrapods, such as chicken and *Xenopus*, but is not apparent with zebrafish. The overall conservation of synteny is belied by multiple types of structural variation unique to the *L. cinereus* lineage (Fig. 3, Supplemental File S11). For example, *L. cinereus* orthologs of the human CECR genes *Pex26*, *Il17ra*, and *Hdhd5* are present in a rearranged order not found in any other species examined. Several genes annotated in *L. cinereus* were not present in at least some of the other bat species included in the study. Three genes appear to have undergone a copy-number expansion in the *L. cinereus* lineage: a *Tbx1* homolog, *Btf3*, and *Tuba8*, although only one gene copy of each is present on scaffold 533 (these expansions are discussed further below). A mixture of conserved synteny for a subgroup of genes and dynamic structural evolution also characterizes the human-mouse comparative alignment (Puech et al., 1997), which, coupled with the frequent clinical observation of genomic rearrangements of 22q11, suggests that the core set of CECR genes are maintained in physical proximity by purifying selection rather than a low rate of structural mutation *per se*. However, some other apparent novelties in *L. cinereus* are actually annotation artifacts on closer inspection (Supplemental File S11). For example, *Atp6v1e1* and *Bid* were not annotated in *L. cinereus*, but homologous sequences were detected by BLASTX searches of the genomic scaffold, whereas other genes putatively unique to *L. cinereus* based on orthogroup clustering are actually fragmented annotations of single genes, leading to orthogroup artifacts.

A third cluster of three positively selected genes on scaffold 111 exhibits partially conserved synteny with diverse tetrapods (Supplemental File S12). The genes are periostin (*Postn*), FRAS1-related extracellular matrix protein 2 (*Frem2*), and proline and serine-rich protein 1 (*Proser1*). A fourth positively selected gene encoding sacsin (*Sacs*) is an additional 3-Mb away on scaffold 111 and exhibits conserved synteny in some but not all lineages (Supplemental File S12). However, in all species examined, *Postn*, *Frem2*, and *Proser1* were retained in the same order and relative orientation, even as other elements of chromosomal synteny were lost. No functional relationship among these four genes was apparent to us.

We used both automated and manual approaches to investigate functions of positively selected genes. The DAVID annotation webtool (Huang et al., 2007) did not identify significant clustering of ontology terms among the 143 mouse orthologs submitted, after correction for multiple testing. This contrasts with some other studies that have detected enrichment of annotation categories in positive selection analyses of various mammalian groups (Kosiol et al., 2008; Hawkins et al., 2019; Beichman et al., 2019). We also manually categorized significant genes based on diverse annotation data within NCBI Gene database entries for mouse and human orthologs (Supplemental File S13), supplemented with literature searches. Of 143 positively selected genes with mouse orthologs, we identified 23 genes (16.2%) with a role in immunity or disease, 17 genes (11.9%) related to metabolic homoeostasis and energetics, 15 genes (10.6%) involved in mitochondrial respiration or biogenesis, and six genes (4.2%) with behavioral phenotypes. These categorizations were based on *post hoc* assessments of commonalities that in our judgement show particular relevance to hoary bat ecology. However, as a uniform process was not applied *a priori* to all tested orthogroups, no enrichment test can be performed for these manual classifications.

### Selected genes with immune function and identification of a Steep1 paralog

Immune function was the most prominent theme among selected genes (Supplemental File S13), consistent with patterns of selection on mammal genes generally (Kosiol et al., 2008) and bats specifically (Zhang et al., 2013; Hawkins et al., 2019; Jebb et al., 2020). While most listed genes are directly related to pathogen detection and immune response, some were included that are only indirectly related, in that their protein products are known to be co-opted by pathogens during infection (*e.g*., *Hnrnpul1*, *Nxf1*, *Eif5b*). We also note that genes classified as relating to immune processes often have diverse additional functions. For example, immune functions have been ascribed to selected genes involved in phosphatidylinositol signaling (*Pik3r5*, *Pik3ap1*, and *Myo10*) and inositol triphosphate signaling (*Dapp1* and *Plcb2*), yet both signaling pathways regulate cellular homeostasis generally (Koyasu, 2003; Hogan, Lewis & Rao, 2009). Nonetheless, several commonalities exist among these immune-classified genes that support their biological relevance as a group. At least four genes regulate or are regulated by interferons, including *Josd1* (Wang et al., 2017), *Psmb9* (Kataoka et al., 2021), *Usp21* (Chen et al., 2017), and *Dapp1* (Onyilagha et al., 2015). Six genes are involved in surface antigen processing and signaling, including the aforementioned *Psmb9* as a component of the immunoproteasome that processes MHC Class I antigens (Singal, Ye & Quadri, 1995). *B4gat1* is involved in synthesizing poly-N-acetyllactosamine, which contributes to diverse glycoprotein structures including antigens (Ujita et al., 1999; Lee, Kohler & Pfeffer, 2009), *Lrmp* is involved in antigen processing within immune cells (Snyder et al., 1997), *Chst2* is involved in L-selectin mediated leukocyte adhesion (Li et al., 2001), and *Itgb2* encodes a major leukocyte adhesion protein (Arnaout, 1990). *Cul7* negatively regulates two somatic mechanisms of immunoglobulin diversification in B lymphocytes, somatic hypermutation and recombinant class-switching (Luo et al., 2019). The previously mentioned *Dapp1* mediates antigen-specific conjugation of B and T cells in mice (Al-Alwan et al., 2010). We speculate that the energetic demands of migration, hibernation, and solitary roosting behavior on hoary bats favor altered immunoresponse dynamics in lasiurine bats compared with ancestral Vespertilionids.

**Figure 4:**
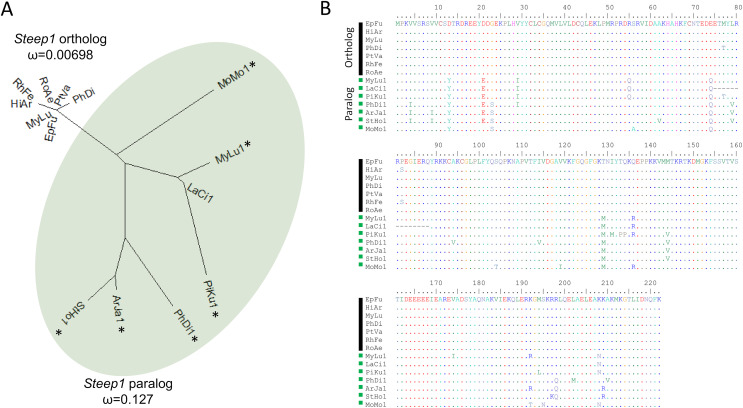
A derived *Steep1* paralog is positively selected within Yangochiroptera. (A) Neighbor-joining dendrogram of the Steep1 ortholog together with the Steep1 paralog (shaded in green), with distances based on nonsynonymous amino-acid substitutions only. The evolutionary rate parameter ω computed under PAML Model 0 is shown for each group of orthologous sequences. The likelihoods of PAML Model 2 were computed with and without branch labels denoting the Steep1 paralog as the foreground branch. The labeled model was significantly more likely than the unlabeled model or Model 1. (B) A protein alignment of bat Steep1 orthologs and the novel paralogous sequences in Yangochiroptera. Four-letter species codes for bats are: EpFu = *Eptesicus fuscus*, MyLu = *Myotis lucifugus*, HiAr = *Hipposideros armiger*, RhFe = *Rhinolophus ferrumequinum*, RoAe = *Rousettus aegyptiacus*, PtVA = *Pteropus vampyrus*, PhDi = *Phyllostomus discolor* [*Steep 1* paralog clade] MoMo = *Molossus molussus*, MyLu = *M. lucifugus*, Laci = *Lasiurus cinereus*, PiKu = *Pipistrellus kuhlii*, PhDi = *P. discolor*, ArJa = *Artibeus jamaicensis*, StHo = *Sturnira hondurensis*.

We initially identified *Steep1* as another immunity gene under positive selection. *Steep1* encodes a transport chaperone of STING, a critical regulator of interferon expression that responds to signatures of viral infection (Ishikawa, Ma & Barber, 2009; Holm et al., 2012; Härtlova et al., 2015; Liu et al., 2019b). The putative *Steep1* ortholog of *L. cinereus* had seven unique amino-acid substitutions among 208 aligned positions, whereas the other bat species were invariant except at gapped (masked) positions. However, additional BlastP searches showed that the *L. cinereus* sequence is actually paralogous to *Steep1* and thus the initial selection test was invalid. These homology searches identified a novel paralog within suborder Yangochiroptera only that is orthologous to *Ensembl*-annotated gene ENSMLUG00000026380. Although the coding sequence of this *M. lucifugus* gene occurs on a single exon, suggesting a retrogene, an initial noncoding exon is annotated and supported by RNAseq evidence in the Gene database entries of all putative orthologs we identified (Fig. 4, Supplemental File S14).

While gene duplication is a common evolutionary occurrence, the potential for alternative regulation of STING *via* a *Steep1* paralog is of particular interest given that STING is a powerful mediator of innate immunity, particularly to viruses (Clayton & Munir, 2020), and one that has evolved dampened activity in bats (Xie et al., 2018). Indeed, human symptoms associated with infection by the coronavirus SARS-CoV-2 have been characterized as a detrimental STING response (Berthelot & Lioté, 2020). We therefore performed a revised test of positive selection in which we labeled the duplicated orthologous group as the foreground branch with the ancestral *Steep1* as the background (Fig. 4). The resulting test of diversifying selection acting on the duplicated *Steep1* was highly significant (*P* = 6.95E−9).

Interestingly, a processed retrogene of *Steep1* is present in both human (ENSG00000233170) and mouse (ENSMUSG00000048188), of independent origins based on their genomic locations and absence in sister taxa. The human ‘pseudogene’ lies within an intron of *Erlin2* in both Old World and New World monkeys (*e.g*., ENSANAG00000019514) but is absent in the tree shrew genome (Supplemental Figure S15A). While it is beyond the scope of this study to review annotation evidence in all relevant genomes, a survey of representative New World and Old World primates (Supplemental Figure S15B) reveals that the derived ‘pseudogene’ on human chromosome 8 is well conserved despite a presumed origin circa 50 Mya, based on molecular clock dating of New and Old World primate divergence (Perez et al., 2013). As with the paralog in Yangochiroptera, Gene database entries suggest that the primate *Steep1* retrogene is transcribed above the background level of surrounding intron sequence (Supplemental Figure S15A, https://www.ncbi.nlm.nih.gov/gene/666594). The functions and evolutionary dynamics of retained *Steep1* retrogenes may collectively merit further exploration.

### Energetics

Bats have very high metabolic rates, primarily due to the energetic demands of flight (O’Shea et al., 2014) and positive selection on cellular respiration genes has previously been shown for bats as a whole relative to other mammals (Shen et al., 2010). *L. cinereus* is further challenged by its extreme migratory behavior and seasonality of habitat and of prey. It is reasonable to suspect that innovations related to heterothermy, torpor, hibernation, and migratory capacity (*e.g*., Davis, 1970; Cryan & Wolf, 2003; Geiser, 2013; McGuire, Fenton & Guglielmo, 2013; Weller et al., 2016) would entail selective pressure on genes related to energetics and metabolic homeostasis.

Of the 17 positively selected genes we identified relating to lipid metabolism and energetics (Supplemental File S13), at least five have roles in regulating adipogenesis. Mesenteric estrogen dependent adipogenesis (*Medag*) is adipogenic (Zhang, Chen & Sairam, 2012) whereas zinc-finger transcription factor *Zfp521* is a negative regulator of adipocyte differentiation from stem cells (Chiarella et al., 2018). Subunit *Med23* of the Mediator Complex is directly required to transduce insulin signaling so that adipocyte differentiation can be initiated by *Krox20* (Wang et al., 2009). *Mtch2* promotes both adipogenesis (Jiang et al., 2019) and lipid accumulation (Bar-Lev et al., 2016) and is strongly expressed in white adipocytes (Kulyté et al., 2011). Genetic variation in *Mtch2* has been linked to human obesity (Kulyté et al., 2011). *Mtch2* is particularly of interest with regard to hibernation physiology, as differences in *Mtch2* transcript or protein abundance are associated with hibernation states of brown bear (*Ursos arctos*) (Chazarin et al., 2019) and ground squirrel (*Ictidomys tridecemlineatus*) (Ballinger et al., 2016). A combined transcriptomic – proteomic approach associated *Zfp512* abundance with increased intramuscular fat deposition and adipogenesis in pigs (Ma et al., 2020).

At least eight positively selected genes have roles in modulating the sensitivity of differentiated cells to metabolic hormones (Supplemental File S13). Regulatory factor X6 (*Rfx6)* has strongly biased expression in K cells and regulates their secretion of gastric inhibitory protein, thereby increasing insulin sensitivity and lipid accumulation (Suzuki et al., 2013). SH2B adaptor protein 1 (*Sh2b1)* is a potent determinant of fat accumulation that is responsive to leptin signaling; *Sh2b1* deletion in mice reduces brown fat function and alters body temperature (Jiang et al., 2020). Rap guanine exchange factor *Rapgef4* mediates cyclic AMP stimulation of insulin secretion by pancreatic β-cells (Song et al., 2013; Henquin & Nenquin, 2014) whereas deletion of replication initiator 1 (*Repin1*) promotes insulin sensitivity *via* glucose stimulation (Kunath et al., 2016). Transcription factor *E2f8* promotes gluconeogenesis and when overexpressed impairs insulin sensitivity in hepatocytes (Chen et al., 2019). *Perlecan* inhibits adipocyte growth, reduces insulin sensitivity, and promotes oxidative muscle fiber development (Yamashita et al., 2018). In cultured islet cells, *Gjb4* inhibits proliferation, insulin secretion, and apoptosis, and the gene is highly expressed in a mouse model of obesity (NZO), in contrast to the leptin-deficient obesity strain B6-ob/ob (Gässler et al., 2020). Deletion or inhibition of phosphodiesterase 10A (*Pde10a*) limits the onset of diet-induced obesity and insulin resistance in mice (Nawrocki et al., 2014).

Three positively selected genes encode lipid metabolizing enzymes or proteins that directly modulate their activity. Phospholipid phosphatase 1 (*Plpp1*) encodes a membrane-bound protein that supports uptake of lipids from the extracellular space (Brindley & Waggoner, 1998), lipase maturation factor 1 (*Lmf1*) is required for the correct folding of certain lipases including hepatic lipase and lipoprotein lipase, but also has a more general role in lipid homeostasis in relation to redox stress as mentioned previously (Ehrhardt, Bedoya & Péterfy, 2014). *Etfbkmt* is a methyltransferase that alters the activity of the ‘hub’ protein ETF-β (Rhein et al., 2014), which as a heterodimer with ETF-α delivers electrons from diverse donors to the membrane-bound electron transport chain (Henriques et al., 2021). The ETF heterodimer controls the flux of fatty-acid oxidation and may modulate energy production during fasting or otherwise when fatty-acid oxidation is upregulated.

Two positively selected genes were involved in the behavioral regulation of metabolism by the hypothalamus. The ligands of neuropeptides B/W receptor 1 (*Npbwr1*) strongly affect feeding behavior of mice in a photoperiod-dependent manner (Tanaka et al., 2003). Male mice that were *Npbwr1*-null exhibited adult-onset obesity that increased with age and when fed a high-fat diet, were hyperphagic, and had decreased locomotor activity (Ishii, Fei & Friedman, 2003). Plasma levels of glucose, leptin, and insulin were all elevated in these males whereas female nulls did not show metabolic defects. Single-minded homolog (*Sim1*) is essential for the developing paraventricular nucleus of the hypothalamus (Michaud et al., 1998) and experimental *Sim1* deficiency, conditional knockout, and ablation of *Sim1* neurons all cause altered feeding behavior (Holder et al., 2004; Xi et al., 2012; Tolson et al., 2014). Genetic variation at the *Sim1* locus is associated with human obesity risk as well (Ramachandrappa et al., 2013; Liu et al., 2019a).

Fifteen genes associated with mitochondria were positively selected, some of which overlap with the lipid homeostasis genes already described, which is to be expected given the central role of mitochondria in energy production. Selected genes encoding mitochondrial membrane transporter components include *Slc25a1*, *Slc25a18*, *Mtch2*, and *Fam173a*, whereas selected genes encoding electron transport proteins include *Cyc1*, *Uqcrfs1*, *NdufA12*, *NdufB10*, and *Etfbkmt*. Genes important for mitochondrial maintenance include *Peo1* (Sarzi et al., 2007), *Prorp* (Howard et al., 2012), *Pum2* (D’Amico et al., 2019), and *Capn3*. *Capn3* mutations cause heterogeneous forms of autosomal recessive limb-girdle muscular dystrophy in humans, due in part to abnormal mitochondrial biogenesis in skeletal muscle (Kramerova et al., 2009; Yalvac et al., 2017).

### Sensory and behavioral gene functions

Perhaps surprising given previous results (Kosiol et al., 2008; Jebb et al., 2020), we did not identify positively selected genes with unambiguous roles in sensory perception, although a strong candidate is *Chrna9*. This gene encodes the α9 nicotinic cholinergic receptor subunit, which is strongly expressed in sensory hair cells of the ear (Scheffer et al., 2015; Liu et al., 2018). The gene has been studied in the contexts of auditory stimulus modulation by cochlear hairs (*e.g*., Boero et al., 2020) and maintenance of orientation and balance by vestibular hairs (*e.g*., Poppi et al., 2018). For example, analyses of mouse mutants with altered or lost function suggest that *Chrna9* modulates the “gain” (stimulus strength) of various auditory signals by altering cochlear sensitivity, including sensitivity to sound frequency, to persistent background noise, and to extreme noise (Taranda et al., 2009; Wedemeyer et al., 2018; Boero et al., 2020). We find selection on *Chrna9* intriguing given the challenges to sensory discrimination associated with echolocation (Corcoran & Moss, 2017) and the recent identification of a novel microcall form of echolocation by *L. cinereus* (Corcoran & Weller, 2018). However, *Chrna9* is expressed in diverse mammalian tissues and has also been implicated in the perception of pain (Vincler et al., 2006) and psychological stress response (Mohammadi, Burton & Christie, 2017). Whether positive selection on *Chrna9* is related to sensory hair function in *L. cinereus* therefore remains to be determined. Additionally, acetylcholine receptor subunits form diverse heteromeric combinations that have distinct properties (Millar, 2003), such that the phenotypic significance of the *L. cinereus Chrna9* sequence may depend on the particular channel complex in which it is constituted.

**Figure 5:**
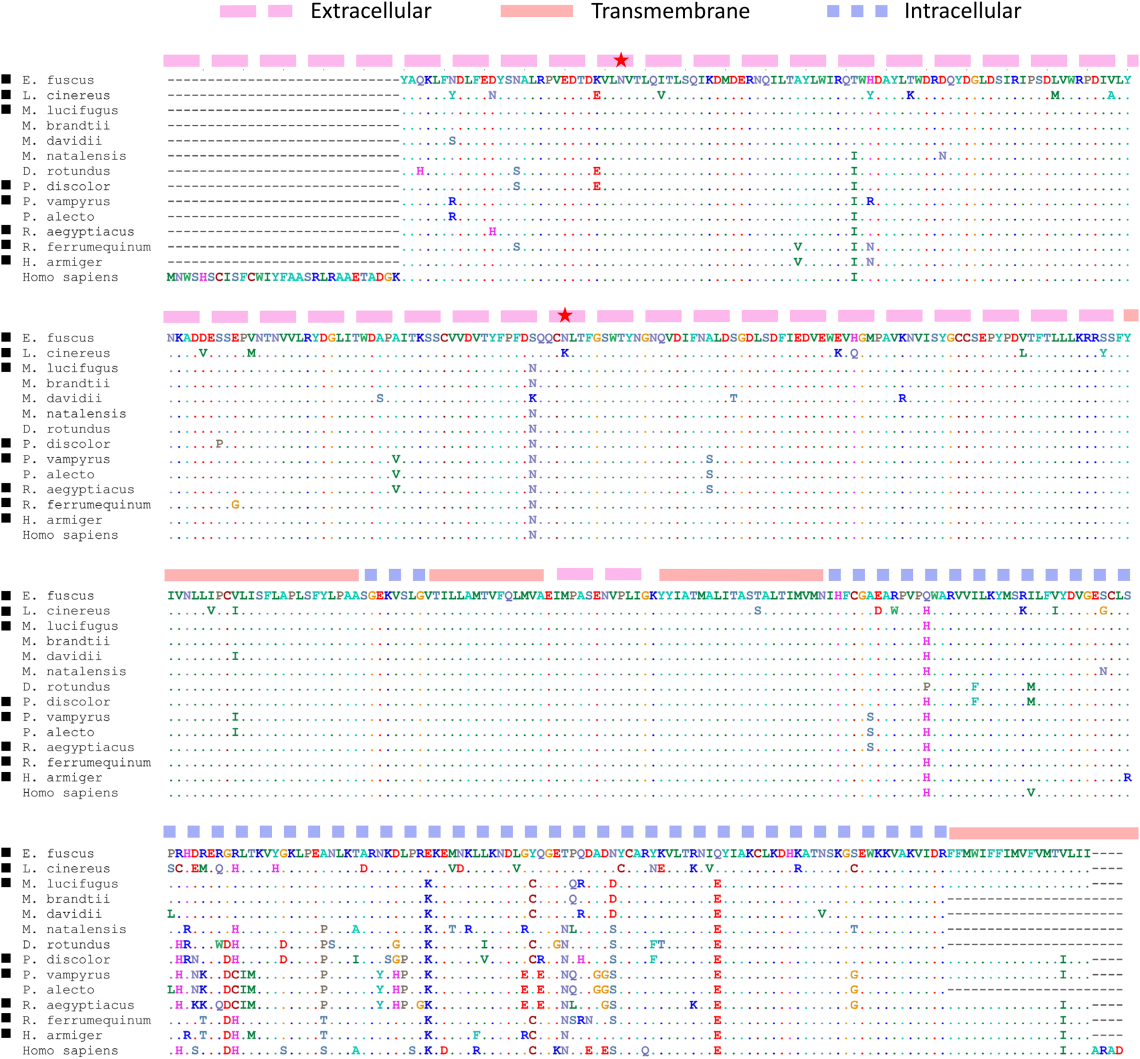
Protein substitutions unique to the *L. cinereus* Chrna9 gene relative to protein structure and functional sites. Black squares denote bat sequences included in the selection test, with additional bat sequences and the human sequence also included for comparison. Alignment coordinates are shaded orange, pink, or blue according to the secondary structure predicted by TMHMM (Krogh et al., 2001). Gaps occur at each end of the alignment because the tested orthogroup alignment was shorter than the full human protein sequence, for which glycosylation sites (red stars) are annotated in the corresponding NCBI accession record. The second glycosylation site has been abolished by an N–>K amino acid change in the *L. cinereus* sequence. See text for additional details.

Several radical amino-acid changes unique to the *L. cinereus* CHRNA9 protein (Fig. 5) occur in the extracellular N-terminal region that contains the acetylcholine binding loops (Taly et al., 2009) and an otherwise conserved glycosylation site is uniquely abolished in this region (the substitution was confirmed by inspecting raw reads aligned to the reference). Glycosylation sites are important both for cellular trafficking during protein maturation and for assembly of multimeric complexes, but it is difficult to predict the effects of their abolishment (Wanamaker & Green, 2005). Substitutions in the extracellular region of CHRNA9 have been shown to alter calcium permeability of α9α10 cholinergic receptors in mammalian cochlear hair cells (Lipovsek et al., 2014), although the two mutations studied in that work are not evident in *L. cinereus* or in bats generally (Fig. 5). Importantly, the long intracellular spacer region linking transmembrane domains is highly variable among bat species, likely due to reduced functional constraint, potentially biasing the selection test. We therefore repeated the PAML selection test on the N-terminal extracellular region only and confirmed a significant result (*P* = 0.00089, ω_2_ = 1.000). Functional analyses (cf. Lipovsek et al., 2014; Marcovich et al., 2020) of bat *Chrna9* haplotypes could be employed to potentially identify divergent properties of *L. cinereus* sequences.

Some of the positively selected genes we identified have documented behavioral phenotypes in human or mouse, including impacts on diurnality, feeding behavior, and social interactions. For example, polymorphisms of the largely uncharacterized gene *D430041D05Rik* were associated with diurnal preference in a human twin study (Melroy-Greif et al., 2017) and had previously been associated with suicidal tendencies in bipolar patients (Willour et al., 2012). The *Timeless* gene encodes a circadian clock component; a nonsense mutation alters photic entrainment and causes familial advanced sleep phase – a shifting forward of diurnal activity but with normal photoperiod – in human and mouse (Kurien et al., 2019). Several positively selected genes affecting metabolic homeostasis are also linked to behavioral phenotypes when manipulated, including the genes *Sim1* and *Npbwr1* that were discussed above in the context of behavioral control of energy balance. *Sim1* expressing neurons enable normal melanocortin-4-associated sexual behaviors in male mice (Semple & Hill, 2018) and linked genetic variants are associated with erectile dysfunction in human (Jorgenson et al., 2018). *Npbwr1* knockdowns altered stress-response behaviors and circadian rhythm in mice, including prolonged and aggressive interactions with unknown “intruder” mice (Nagata-Kuroiwa et al., 2011). A human study associated a variant *Npbwr1* allele with altered perceptions of, and reactions to, angry facial expressions (Watanabe et al., 2012). In addition to effects on insulin sensitivity, *Rapgef4*-deficient mice exhibit reduced social interactions and ultrasonic vocalizations but no impairment of memory, learning, or nonsocial behaviors (Srivastava et al., 2012). *Pde10a* is highly expressed in neurons of the striatum (Seeger et al., 2003), which are important for limb coordination and motivated learning, and *Pde10a* knockdown impairs motivated learning in mice *via* both positive and negative stimuli (Piccart et al., 2011). Collectively, these behavioral phenotypes are intriguing with respect to the ecology of hoary bats, which are conspicuous in roosting predominantly in isolation (Shump & Shump, 1982), exhibiting sex biased migration that may relate to divergent thermoregulatory strategies (Cryan & Wolf, 2003; McGuire, Fenton & Guglielmo, 2013), and frequent use of facultative torpor during migration (McGuire, Jonasson & Guglielmo, 2014). More speculatively, circadian rhythm and patterns of exploratory or anxious movement are believed to contribute to migratory propensity in birds (Ramos, Delmore & Liedvogel, 2017 and references therein) and could have similar effects in bats.

### Genes associated with craniodysmorphy

A remarkable number of positively selected genes in *L. cinereus* have orthologs in human associated with craniodysmorphy when deleteriously mutated. They include five genes associated with the CECR and DGCR regions mapped on human 22q11. Cat-eye syndrome is a dosage-sensitive syndrome arising from segmental or supernumerary duplication (Footz et al., 2001). While defects are congenital, individuals may be developmentally mosaic for the chromosomal duplication and thus phenotypic penetrance is highly variable. Mild craniofacial dysmorphy, particularly affecting the eyes and ears, is characteristic of the syndrome but other conditions include heart and renal defects and anal fistula. Of the five positively selected genes syntenic with the CECR, *Slc25A18* and *Cecr2* are most strongly implicated in disease etiology. *Slc25A18* is a mitochondrial membrane transport channel and *Cecr2* contains a bromodomain associated with chromatin-based transcriptional regulation. Additional positively selected genes associated with craniodysmorphy include *Frem2*, which underlies the cryptopthalmy and syndactyly of Fraser syndrome (Van Haelst et al., 2008; Zhang et al., 2019a), *Perlecan*, which is implicated in Schwarz–Jampel syndrome (Stum et al., 2006), and *Cul7*, a cause of 3M syndrome (Huber et al., 2005). These human syndromes may ultimately reflect developmental pleiotropy (Lindsay et al., 2001), *i.e*., impacts on tissues that arise from a common primordium. Our findings most likely signal developmental novelty affecting the embryonic pharyngeal arch that gives rise to thymus, heart, and head, which would be consistent with the expression biases shown in Fig. 1.

### Expansion of a Tbx1-like gene in *L. cinereus*

As mentioned previously, orthogroup copy numbers in the CECR region (Supplemental File S11) initially suggested expansions of the genes *Tbx1*, *Btf3*, and *Tuba8* within the *L. cinereus* lineage. Most copies of these three orthogroups are dispersed in the *L. cinereus* genome and have multiple exons, *i.e*., they are not processed retrogenes or tandem duplicates. However, some annotations are fragmented and we cannot assess the activity or function of individual gene copies from the present data. While a general investigation of copy number evolution in *L. cinereus* is outside the scope of this work, the causal link between *Tbx1* dosage and developmental phenotypes in human and mouse that overlap with CECR and DGCR phenotypes (discussed below) suggests a connection between positive selection and gene amplification in the region. Gene amplification is an important but often ephemeral mechanism of adaptation in response to selection on gene expression levels in bacteria (Tomanek et al., 2020) and invertebrates (Bass et al., 2013) but its role in vertebrates is less clear.

Two of the potentially expanded genes, *Tbx1* and *Tuba8*, are also present on human 22q11, and *Tbx1* dysregulation is considered causal for DiGeorge syndrome (Merscher et al., 2001), whereas *Tuba8* is adjacent to and dysregulated by DGCR haploinsufficiency but not implicated in the syndrome itself (Dantas et al., 2019). In contrast, the functional human ortholog of *Btf3* is located on chromosome 5 and is a basal transcription factor necessary for transcription initiation (Zheng et al., 1990). *Btf3* pseudogenes appear to be common in mammalian evolution as numerous presumed pseudogenes are listed in the Ensembl database for both human and mouse. *Tbx1* is a key transcription factor in many developmental pathways, including cardiac morphogenesis (Alfano et al., 2019) and otocyst (inner ear) development (Raft et al., 2004), and is an antagonist of retinoic acid pathways (Koop et al., 2014). In human, *Tbx1* haploinsufficiency is associated with congenital defects of the heart, hypoplasia of the thymus, hypoparathyroidism, and facial dysmorphia (Du, de la Morena & van Oers, 2020).

Alignment of the *Tbx1* orthogroup sequences revealed that the genes annotated in *L. cinereus* are structurally distinct from *Tbx1* annotated in the other bat species, although a similar homolog is also found in *E. fuscus*. Only the 5′ portion of the *Tbx* domain is present in these “*Tbx1*-like” sequences, and the sequences are shorter than other *Tbx* homologs. BLASTX searches demonstrated that the true *Tbx1* ortholog is in fact present in *L. cinereus* but unannotated on scaffold 536, which is colinear with the *Tbx1*-containing region of *E. fuscus* (Supplemental File S16). A phylogeny of the *Tbx1*-like sequences together with BlastP matches from the other vespertilionid bats studied demonstrates that the novel gene family is not a mis-annotation of expected *Tbx* homologs, and that it is expanded only in the *L. cinereus* lineage (Supplemental File S16). Importantly, both deletion and overexpression of *Tbx1* in mouse generate DiGeorge-like phenotypes in a dose-dependent manner (Liao et al., 2004), suggesting that amplification of the novel *Tbx1*-like gene could also drive a dosage-sensitive phenotype related to the positively selected genes in the region.

### Limitations and caveats of this study

While our annotations are based on a draft genome uninformed by RNA-seq data, the numbers of genes and orthogroups identified were on par with expectation and the number of positively selected genes identified was comparable to some recent studies in bats (Shen et al., 2010; Hawkins et al., 2019). Nonetheless, there are general limitations and biases to identifying positive selection based on excess nonsynonymous mutation alone. Coding-sequence prediction is vulnerable to sequencing error in the underlying genome, which disrupts features such as splicing signals and reading frames, thereby reducing the number of codons available for analysis or introducing alignment errors. On the other hand, sequencing error should not directly bias evolutionary rate estimates because it impacts synonymous and nonsynonymous sites proportionally, and also because the other analyzed genomes are similarly affected. Alignment ambiguity is another major source of uncertainty, particularly in more variable protein regions, resulting in incorrectly inferred codon orthology and substitution rates. Even when the primary sequence is correct and complete, substitution rates must be estimated for abstracted models with simple parameterizations. Excess nonsynonymous substitution as a criterion of positive selection may be inadequate to detect many actual instances of positive selection, in part because the functional significance of accrued amino-acid changes need not be related to their number *per se* (*e.g*., a single nonsynonymous substitution strongly alters STING activity in bats (Xie et al., 2018)), and also because the excess is evolutionarily transient, such that statistical power depends on the particular set of taxa investigated.

Beyond these ascertainment issues, inferring the biological relevance of positively selected genes presents a further challenge. Pairwise divergence between bats and rodents is high (Jebb et al., 2020), such that the transferability of functional annotations from model organisms may be more tenuous than for many other mammals. Studies of mammalian gene function also depend heavily on gross manipulations in mouse and genetic analyses of clinical disorders in human, yet deleterious phenotypes induced by severe alterations of gene expression or sequence may be dissimilar to positively selected phenotypes in nature. Despite these caveats, a number of independent factors support the biological relevance of the genes we identified. While we manually categorized genes after the fact and ascertainment biases likely exist among functional categories (*e.g*., phenotypes relevant to human health are more likely to be identified), the strong representation of genes with immune function or mitochondrial function is consistent with the literature (Kosiol et al., 2008; Shen et al., 2010; Zhang et al., 2013; Hawkins et al., 2019), particularly as it relates to interferon regulation (Zhang et al., 2013; Xie et al., 2018). Furthermore, both biased expression (higher expression in thymus) and biased genomic location (physical clustering) were observed among positively selected genes, with no plausible connection between these variables and false positive rates. Moreover, all three clusters of positively selected genes discussed in the text occur within regions of ancient synteny, further supporting the view that the genomic clustering we detected is biologically relevant and not due to chance.

## Conclusions

Hoary bats migrate vast distances, are challenging to census, and have poorly characterized hibernacula. As a result, their ecology remains poorly understood despite increasing conservation concerns. Here we have used a relatively rapid, low-cost approach to identify candidate targets of positive selection since divergence of the hoary bat lineage from other Vespertilionid groups, suggesting that the present-day ecology of this group rests in part on substantial evolutionary innovation. Functional annotations and expression data for selected genes strengthen the view that immunity and metabolism are important targets in hoary bat evolution. However, the observation that positively selected genes cluster in regions of ancient synteny is to our knowledge unprecedented in vertebrates and suggests that architecturally constrained regions are not precluded from diversifying selection and may even be hotspots of adaptation.

### Future directions

Assemblies and their annotation are inherently progressive and we expect additional data to further refine the *L. cinereus* gene set. We consider the coding sequences used here preliminary, to be superseded by RNA-supported annotations of a chromosome-level assembly (recently made available by DNAZoo (https://www.dnazoo.org) but not presently available from NCBI). In turn, orthogroup delineation can be enhanced by incorporating local genomic synteny in addition to protein similarity, such as was applied by Jebb et al. (2020) to six bat genomes. For individual candidate genes, the underlying primary sequences should be confirmed through manual annotation if necessary and selection tests buttressed with additional phylogenetic representation. The expression of genes that remain strong candidates after this additional vetting should be characterized specifically in *L. cinereus* and background species. While many bat species are not candidates for captive rearing or experimental manipulation, behavioral and physiological tests may sometimes be tractable, as might immune challenges. *In vitro* or *in vivo* tests of protein function may also be feasible in some cases, such as has been used to investigate acetylcholine receptor function (Marcovich et al., 2020).

Future work may move beyond single-copy orthogroups to explore genes with more complicated evolutionary histories as well as the expansion and contraction of multi-gene families. These approaches will benefit greatly from ongoing genome sequencing initiatives for bats (Teeling et al., 2018). In addition to refining the analysis of protein evolutionary rate, other statistical signatures of natural selection can be explored that reveal more recent selection pressures, including population genomic scans for selective sweeps (Pinzari et al., 2020). Resequencing of opportunistically sampled bats obtained from wind-turbine facilities and targeted sampling of congeners and disjunct *L. cinereus* populations would provide a powerful framework for identifying more recent episodes of selection, for example signatures of turbine-imposed selection if such exists. Such strategies might also reveal shifts in the neuroendocrine control of metabolism in island populations with altered seasonality and reduced migration.

## Supplemental Information

10.7717/peerj.13130/supp-1 Supplemental Information 1 Alignment and selection test statistics for each orthogroup. Round 1 values are for all tested orthogroup alignments whereas Round 2 values are after manual review of initially significant tests.

10.7717/peerj.13130/supp-2 Supplemental Information 2 Maker-derived gene models in gff format. The gff format specification is detailed at https://en.wikipedia.org/wiki/General_feature_format and a number of software packages are available to extract or manipulate gff-formatted features.

10.7717/peerj.13130/supp-3 Supplemental Information 3 Orthogroup alignments in FASTA format. Initially tested orthogroups that have been edge-trimmed as described in the Methods.

10.7717/peerj.13130/supp-4 Supplemental Information 4 Manually revised orthogroup alignments. Likely alignment errors have been manually deleted between conserved codons. Recovery of the original codon positions requires alignment to the original accessions in each orthogroup.

10.7717/peerj.13130/supp-5 Supplemental Information 5 Qualitative comparison of PAML and aBSREL *P*-values for branch-specific diversifying selection in the hoary bat (*Lasiurus cinereus*) lineage. (A) PAML and aBSREL P-values sorted by decreasing PAML value. Values are formatted as -log_10_(P). (B) PAML and aBSREL *P*-values sorted by decreasing aBSREL *P*-value. Values are formatted as -log_10_(P). (C) Scatterplots of PAML and aBSREL *P*-values by descending quintile of alignment length (longest alignments in the first panel). Arrows illustrate a qualitative dual-distribution pattern.

10.7717/peerj.13130/supp-6 Supplemental Information 6 Scatterplot illustrating the inverse relation between ω_2_and p_2_estimates for significant genes in the hoary bat (*Lasiurus cinereus*) lineage.

10.7717/peerj.13130/supp-7 Supplemental Information 7 Contingency table and statistics for frequency of positively selected single-exon genes in the hoary bat (*Lasiurus cinereus*) lineage.

10.7717/peerj.13130/supp-8 Supplemental Information 8 Gene expression values for mouse orthologs of tested orthogroups. Mouse orthologs were extracted from the National Center for Biotechnology Information (NCBI) Gene database of the *Myotis lucifugus* gene in each orthogroup. Values are normalized to a maximum of 1.

10.7717/peerj.13130/supp-9 Supplemental Information 9 Top three physical clusters of postively selected *Lasiurus cinereus* genes and their inferred orthologs from model organisms. Orthologies are pre-computed by Ensembl identifier and were extracted from each *Myotis lucifiugu*s page when available, otherwise from the corresponding mouse gene page. Genes are listed in the form “Gene name:chromosme or scaffold:start coordinate:end coordinate:strand”. Colored blocks within species are colinear with the *L. cinereus* orthologs.

10.7717/peerj.13130/supp-10 Supplemental Information 10 Visualization of conserved synteny among mammals of positively selected genes clustered on *Lasiurus cinereus* Scaffold 133. In each pairwise alignment, the same region of human chromosome 16 is compared with mouse, cow, *M. lucifugus*, *L. cinereus*, and zebrafish. Green bars indicate the positions of the human orthologs of the five positively selected *L. cinereus* genes (labeled). Dots represent pairwise protein similarity above threshold values in the same (red) or reverse (blue) orientation. Contiguous strings of protein similarity encompassing multiple genes are interpreted as evolutionary conservation of synteny.

10.7717/peerj.13130/supp-11 Supplemental Information 11 Table of features on Scaffold 533 of the *Lasiurus cinereus* assembly, in the vicinity of the region that is syntenic with the cat-eye critical region (CECR). Features are ordered by coordinates and the orthogroup in which the feature was placed is given, along with the total number of orthogroup elements for each bat species. Green shaded cells highlight perfect or near-perfect orthogroups, whereas yellow shaded cells highlight orthogroups with a high copy number in *L. cinereus*. Features in bold font are landmarks of the human CECR or adjacent DiGeorge critical region (DGCR).

10.7717/peerj.13130/supp-12 Supplemental Information 12 Visualization of conserved synteny among tetrapods of positively selected genes clustered on *Lasiurus cinereus* Scaffold 111. In each pairwise alignment, the same region of human chromosome 16 is compared with frog, chicken, cow, mouse, *Myotis lucifugus*, and *L. cinereus*. Green bars indicate the positions of the human orthologs of the four positively selected *L. cinereus* genes (labeled). Dots represent pairwise protein similarity above threshold values in the same (red) or reverse (blue) orientation. Contiguous strings of protein similarity encompassing multiple genes are interpreted as evolutionary conservation of synteny.

10.7717/peerj.13130/supp-13 Supplemental Information 13 Annotation summaries of positively selected genes, including orthologs, orthogroup properties, expression data, annotation data, and functional classifications. Gene summaries, GeneRIFs, GO terms, and other controlled-vocabulary descriptions of gene function were extracted from the NCBI Gene database. Other annotations drawn from the literature are cited.

10.7717/peerj.13130/supp-14 Supplemental Information 14 Location, structure, and expression of genes inferred to be orthologous to the *Steep1*-like paralog of *Lasiurus cinereus*.. Each image is a genome browser view of the corresponding Gene database entry. The source scaffold is displayed in the upper left corner of each window, with coordinate tracks at the top and bottom of each window. The exon structure of each gene is shown in the first track, with subsequent tracks showing relative RNAseq-based expression and support for individual splicing events. Complete descriptions of track icons and data sources are available from the NCBI Gene database (https://www.ncbi.nlm.nih.gov/gene).

10.7717/peerj.13130/supp-15 Supplemental Information 15 A human *Steep1* ‘pseudogene’ is well conserved and expressed in New World and Old World primates. (A) Location, structure, and expression of the human locus and its orthologs in New and Old World primates. Each image is a genome browser view of the corresponding Gene database entry. The source scaffold is displayed in the upper left corner of each window, with a coordinates track at the base of each window. The exon structure of each gene is shown in the first track, with subsequent tracks showing relative RNAseq-based expression and support for individual splicing events. Complete descriptions of track icons and data sources are available from the NCBI Gene database (https://www.ncbi.nlm.nih.gov/gene). (B) Dendrogram of the *Steep1* paralog in representative primates, illustrating a high-level of protein-sequence conservation.

10.7717/peerj.13130/supp-16 Supplemental Information 16 Expansion of a novel T-box transcription factor (*Tbx*)-like gene family in *Lasiurus cinereus*. (A) Alignment of orthogroup OG0000803 sequences with *Tbx* family homologs of the Vespertilionid species *L. cinereus*, *Eptesicus fuscus*, and *Myotis lucifugus*. The alignment region containing the conserved *Tbx* domain is indicated by the blue box above the alignment, whereas the portion of the domain present in the novel orthogroup sequences is outlined in yellow. (B) Neighbor-joining dendrogram of the alignment in panel A. The two halves of the dendrogram, split here for clarity, are joined on the dotted lines. The left half contains the single *Tbx*-like gene present in *E. fuscus* (XP_027987819) and annotated as *Tbx1-like*, along with the fifteen copies annotated in *L. cinereus*. Not all annotations are complete and whether all are expressed is unknown. The right half of the dendrogram contains all identified *Tbx* homologs with complete domains identified in these three species. Note that an unannotated *Tbx1* ortholog is likely present in *L. cinereus*, as a TBLASTN search with the *E. fuscus* ortholog (XP_028003188) has a strong match in the syntenic genomic location on scaffold 586. The *Tbx* family appears to be approximately complete in *L. cinereus* and shows an approximately 1-to-1 relationship with *M. lucifugus* and *E. fuscus* genes. Therefore, the *Tbx1*-like sequences are not simply incomplete annotation artifacts. The phylogeny used the JTT amino-acid distance measure with pairwise deletion of gaps. Rate variation among sites was modeled as a five-category gamma distribution with a shape parameter of one.

## Additional Information and Declarations

### Data Availability

The following information was supplied regarding data availability: The hoary bat genome sequence that was annotated is available from the ScienceBase repository: Cornman, R.S., Fike, J.M., Oyler-McCance, S.J., and Cryan, P.M., 2021, Genetic variation in hoary bats (*Lasiurus cinereus*) assessed from archived samples: U.S. Geological Survey data release, https://doi.org/10.5066/P9VSG54Z. The gene annotations in gff format and orthogroup sequence alignments in FASTA format are available in the Supplemental Files.
