# Supplementary figures and images for: Positively selected genes in the hoary bat (Lasiurus cinereus) lineage: prominence of thymus expression, immune and metabolic function, and regions of ancient synteny
Source: PeerJ. 2022 Mar 17;10:e13130. doi: 10.7717/peerj.13130 (PMC8934532; doi:10.7717/peerj.13130)

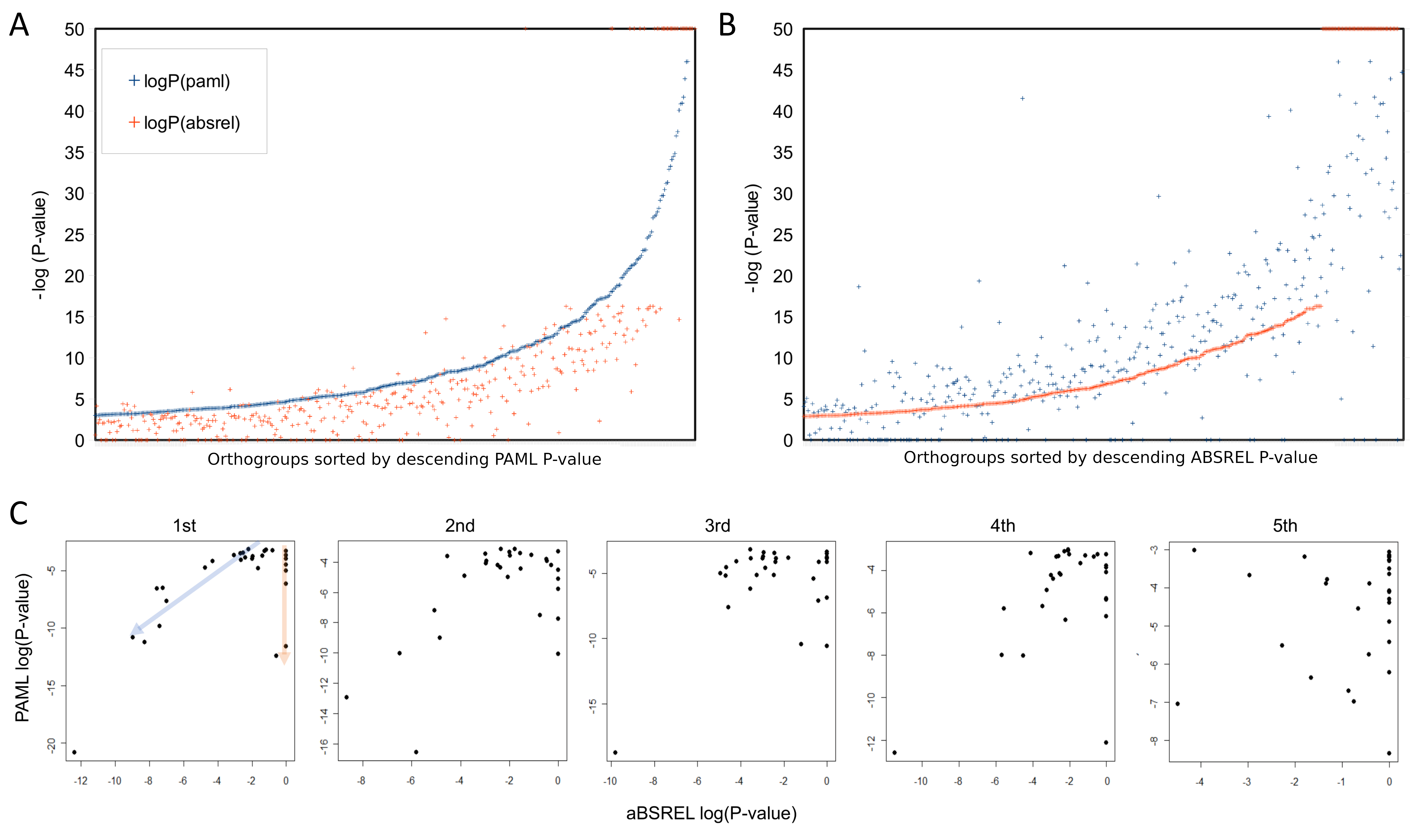

Supplement: Supplemental Information 5 — (A) PAML and aBSREL P-values sorted by decreasing PAML value. Values are formatted as -log10(P). (B) PAML and aBSREL P-values sorted by decreasing aBSREL P-value. Values are formatted as -log10(P). (C) Scatterplots of PAML and aBSREL P-values by descending quintile of alignment length (longest alignments in the first panel). Arrows illustrate a qualitative dual-distribution pattern. [file peerj-10-13130-s005.png]

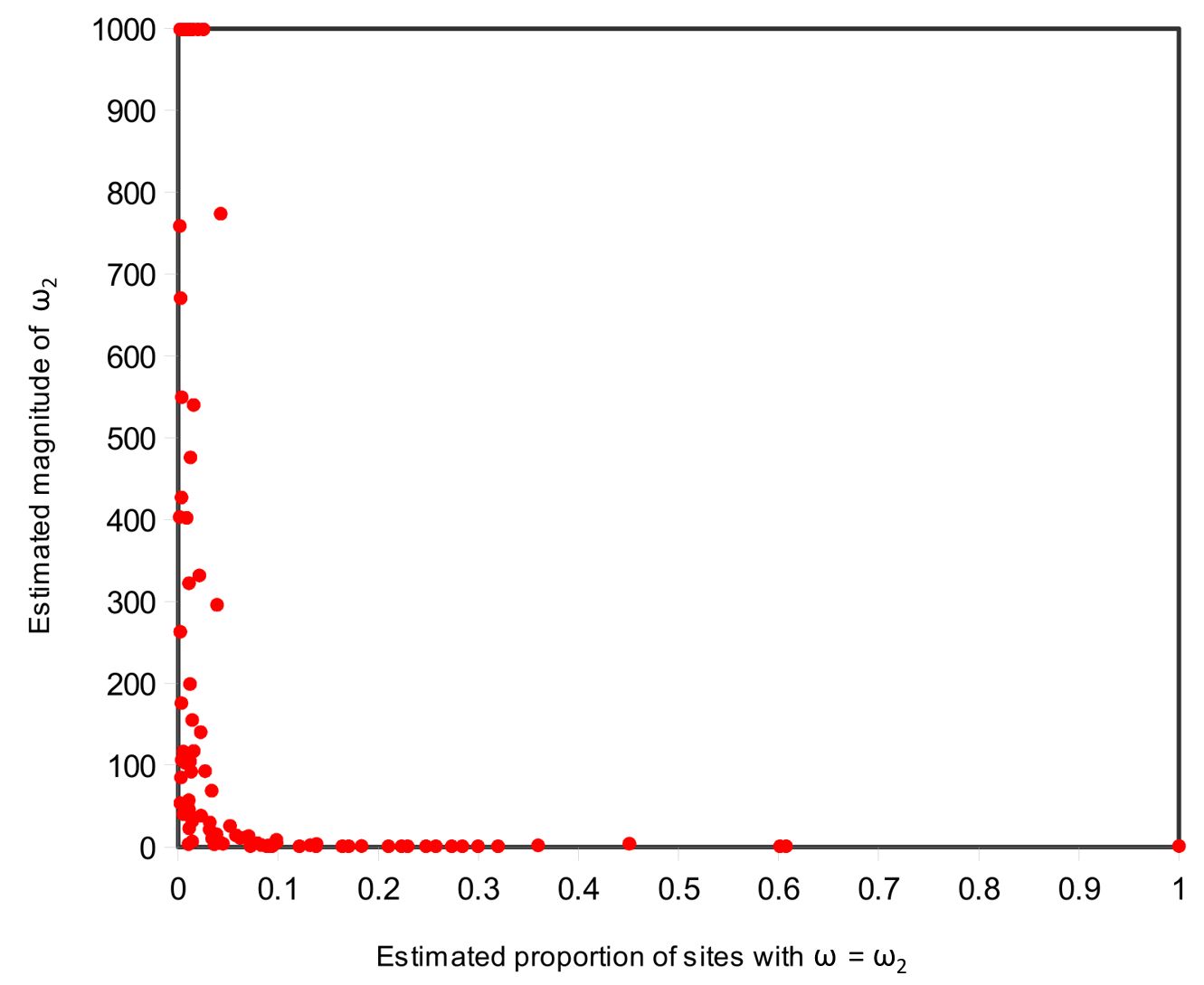

Supplement: Supplemental Information 6 [file peerj-10-13130-s006.png]

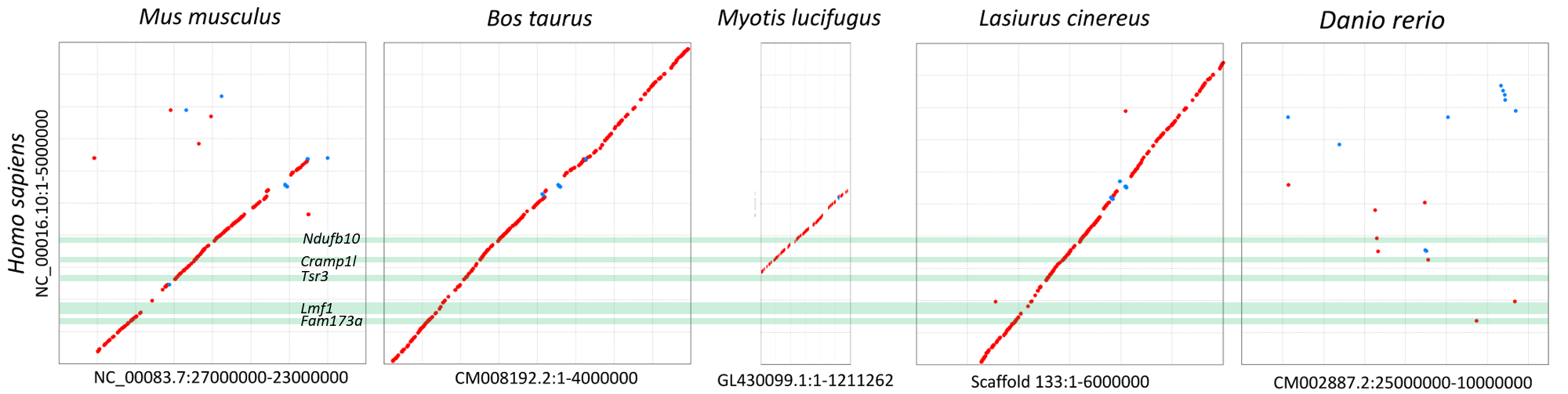

Supplement: Supplemental Information 10 — In each pairwise alignment, the same region of human chromosome 16 is compared with mouse, cow, M. lucifugus, L. cinereus, and zebrafish. Green bars indicate the positions of the human orthologs of the five positively selected L. cinereus genes (labeled). Dots represent pairwise protein similarity above threshold values in the same (red) or reverse (blue) orientation. Contiguous strings of protein similarity encompassing multiple genes are interpreted as evolutionary conservation of synteny. [file peerj-10-13130-s010.png]

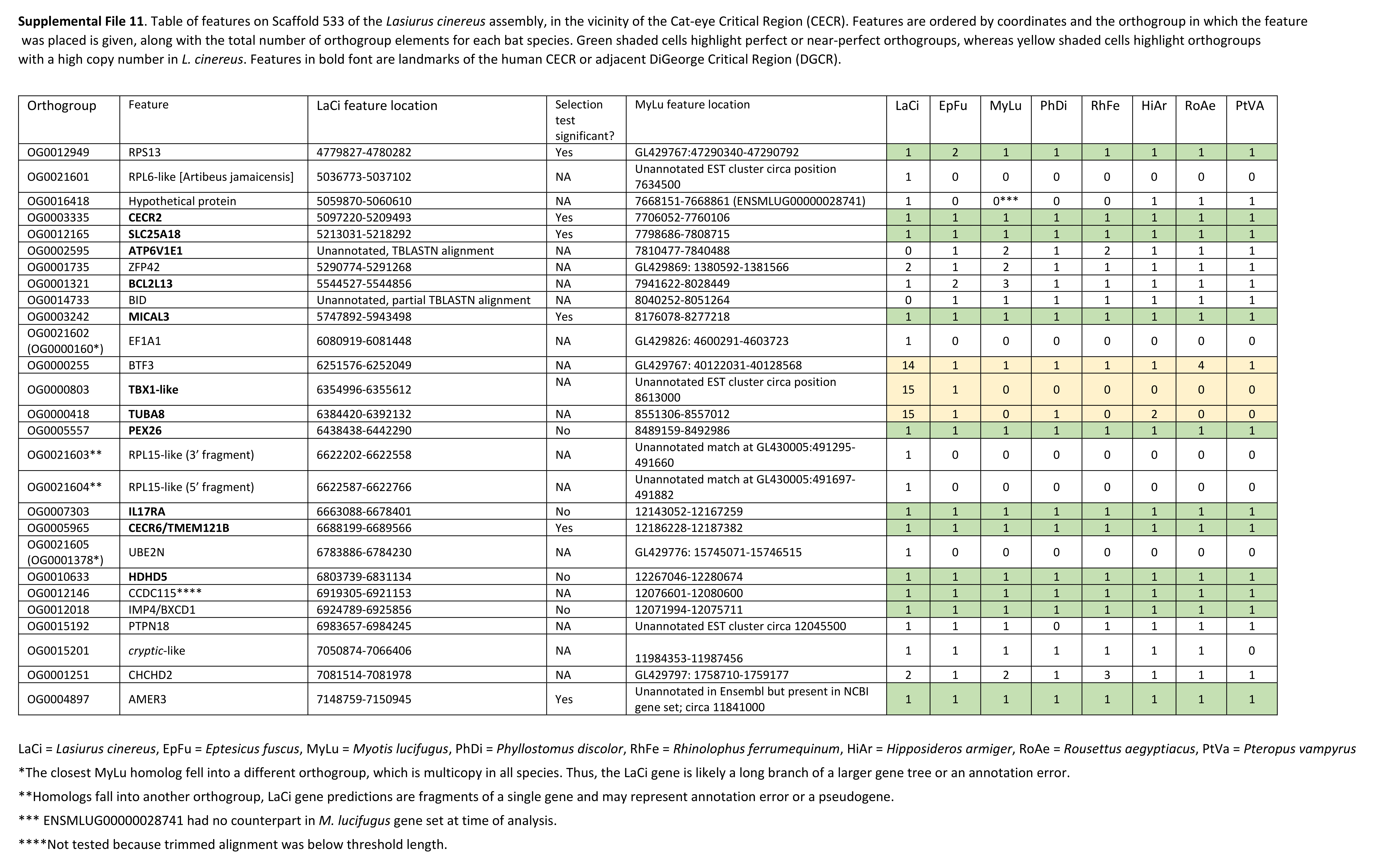

Supplement: Supplemental Information 11 — Features are ordered by coordinates and the orthogroup in which the feature was placed is given, along with the total number of orthogroup elements for each bat species. Green shaded cells highlight perfect or near-perfect orthogroups, whereas yellow shaded cells highlight orthogroups with a high copy number in L. cinereus. Features in bold font are landmarks of the human CECR or adjacent DiGeorge critical region (DGCR). [file peerj-10-13130-s011.png]

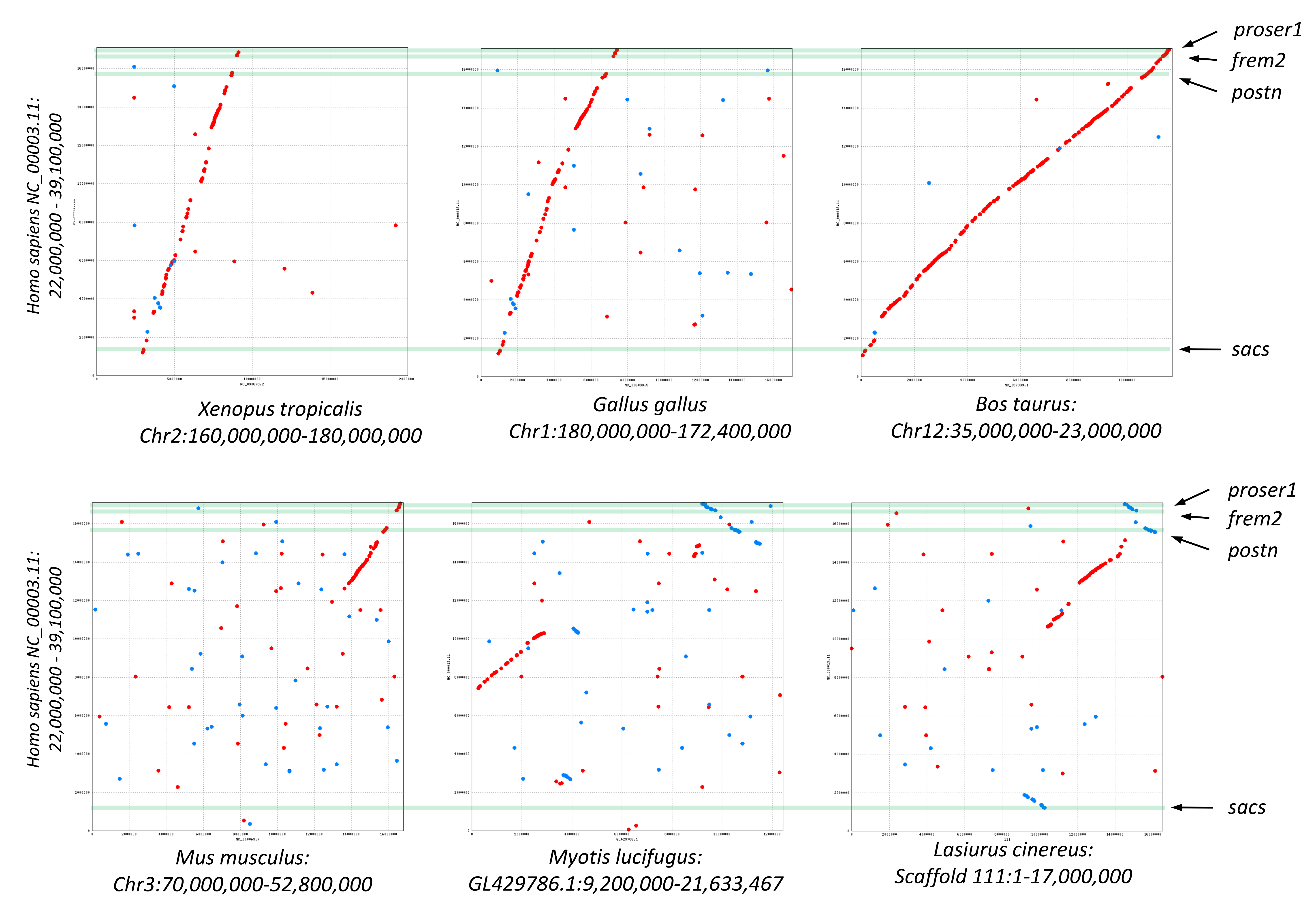

Supplement: Supplemental Information 12 — In each pairwise alignment, the same region of human chromosome 16 is compared with frog, chicken, cow, mouse, Myotis lucifugus, and L. cinereus. Green bars indicate the positions of the human orthologs of the four positively selected L. cinereus genes (labeled). Dots represent pairwise protein similarity above threshold values in the same (red) or reverse (blue) orientation. Contiguous strings of protein similarity encompassing multiple genes are interpreted as evolutionary conservation of synteny. [file peerj-10-13130-s012.png]

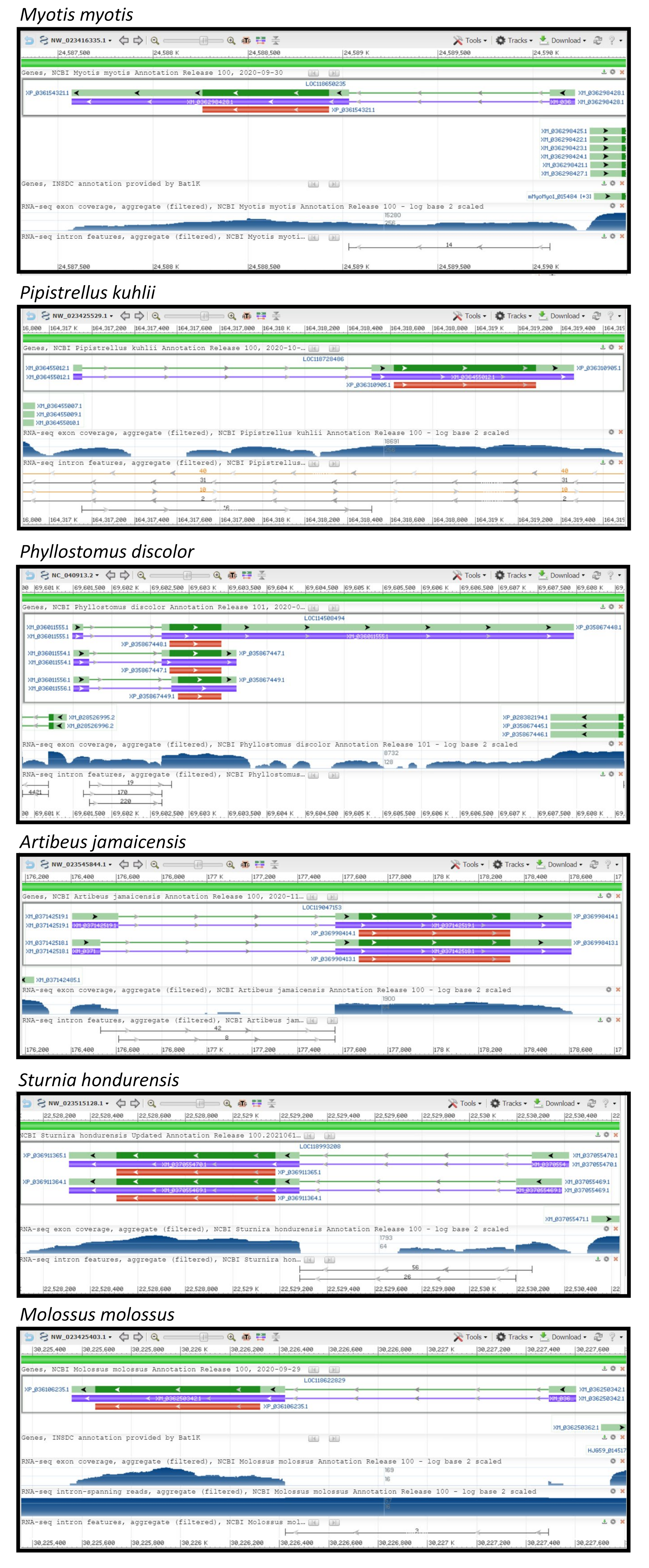

Supplement: Supplemental Information 14 — Each image is a genome browser view of the corresponding Gene database entry. The source scaffold is displayed in the upper left corner of each window, with coordinate tracks at the top and bottom of each window. The exon structure of each gene is shown in the first track, with subsequent tracks showing relative RNAseq-based expression and support for individual splicing events. Complete descriptions of track icons and data sources are available from the NCBI Gene database (https://www.ncbi.nlm.nih.gov/gene). [file peerj-10-13130-s014.png]

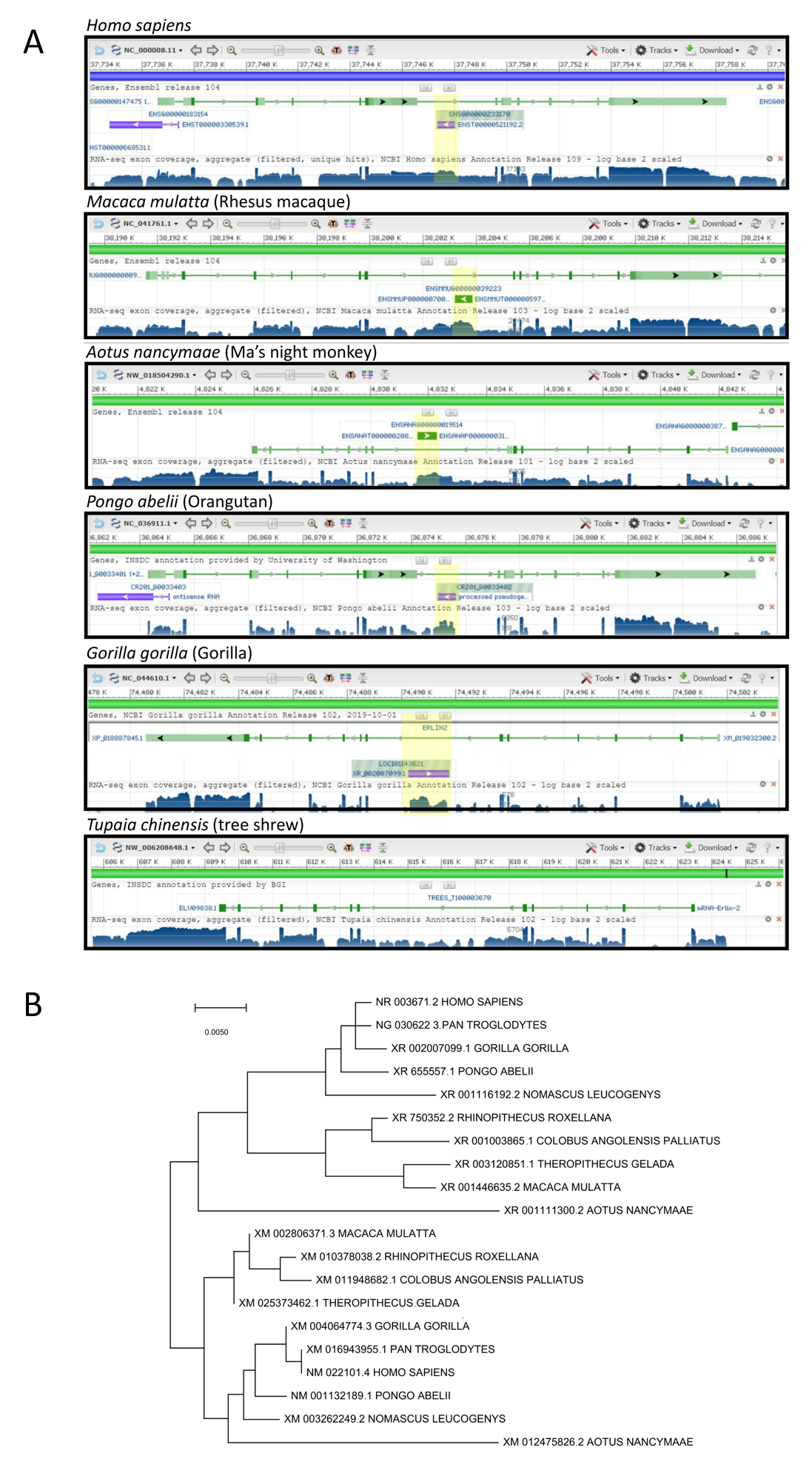

Supplement: Supplemental Information 15 — (A) Location, structure, and expression of the human locus and its orthologs in New and Old World primates. Each image is a genome browser view of the corresponding Gene database entry. The source scaffold is displayed in the upper left corner of each window, with a coordinates track at the base of each window. The exon structure of each gene is shown in the first track, with subsequent tracks showing relative RNAseq-based expression and support for individual splicing events. Complete descriptions of track icons and data sources are available from the NCBI Gene database (https://www.ncbi.nlm.nih.gov/gene). (B) Dendrogram of the Steep1 paralog in representative primates, illustrating a high-level of protein-sequence conservation. [file peerj-10-13130-s015.png]

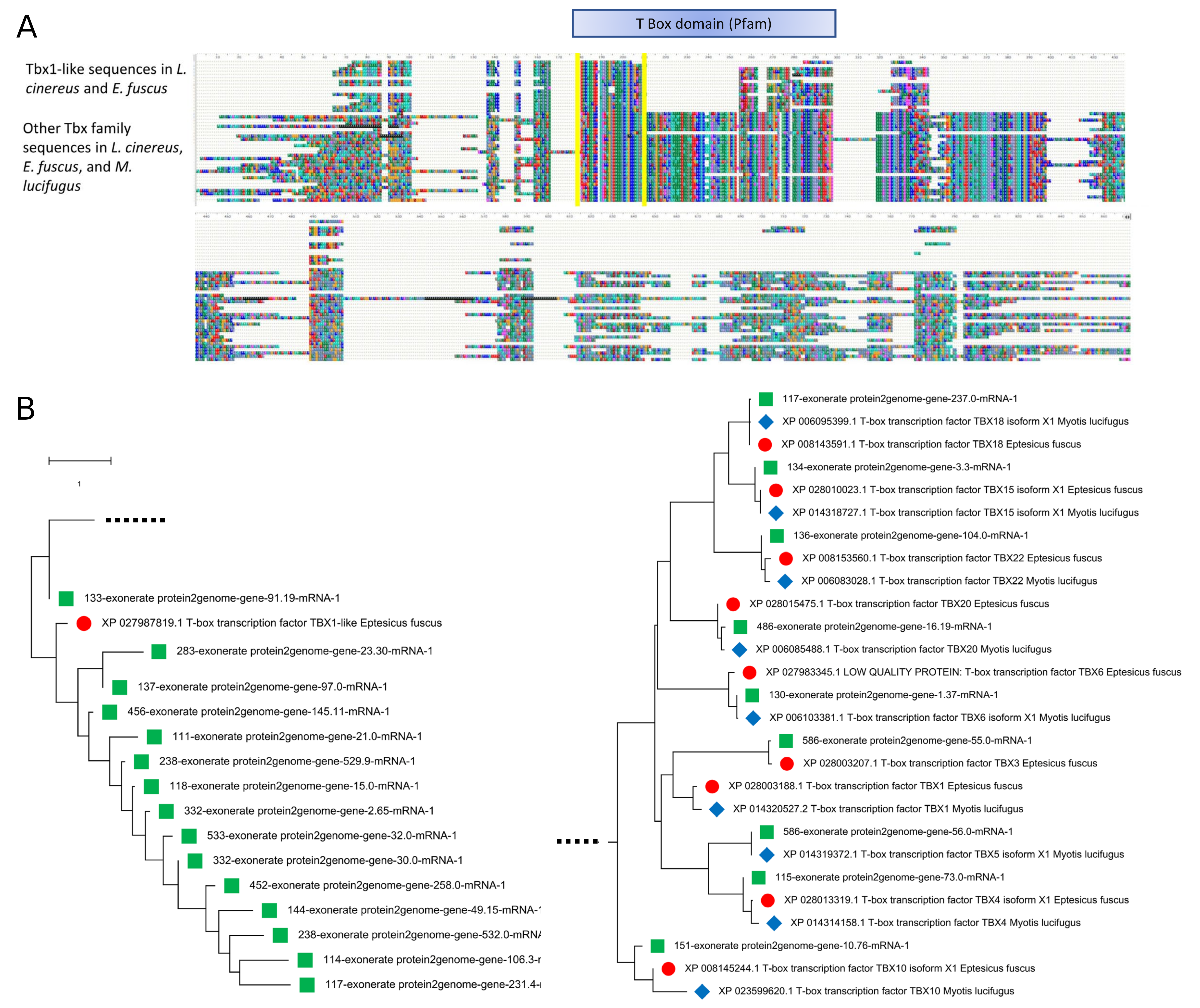

Supplement: Supplemental Information 16 — (A) Alignment of orthogroup OG0000803 sequences with Tbx family homologs of the Vespertilionid species L. cinereus, Eptesicus fuscus, and Myotis lucifugus. The alignment region containing the conserved Tbx domain is indicated by the blue box above the alignment, whereas the portion of the domain present in the novel orthogroup sequences is outlined in yellow. (B) Neighbor-joining dendrogram of the alignment in panel A. The two halves of the dendrogram, split here for clarity, are joined on the dotted lines. The left half contains the single Tbx-like gene present in E. fuscus (XP_027987819) and annotated as Tbx1-like, along with the fifteen copies annotated in L. cinereus. Not all annotations are complete and whether all are expressed is unknown. The right half of the dendrogram contains all identified Tbx homologs with complete domains identified in these three species. Note that an unannotated Tbx1 ortholog is likely present in L. cinereus, as a TBLASTN search with the E. fuscus ortholog (XP_028003188) has a strong match in the syntenic genomic location on scaffold 586. The Tbx family appears to be approximately complete in L. cinereus and shows an approximately 1-to-1 relationship with M. lucifugus and E. fuscus genes. Therefore, the Tbx1-like sequences are not simply incomplete annotation artifacts. The phylogeny used the JTT amino-acid distance measure with pairwise deletion of gaps. Rate variation among sites was modeled as a five-category gamma distribution with a shape parameter of one. [file peerj-10-13130-s016.png]
